# Rare event ABC-SMC$^{2}$

**DOI:** 10.1007/s11222-026-10979-6

**Published:** 2026-09-27

**Authors:** Ivis Kerama, Thomas Thorne, Richard G. Everitt

**Affiliations:** 1 Department of Economics, University of Exeter, Rennes Drive, Exeter, EX4 4PU UK; 2 Computer Science Research Centre, University of Surrey, Guildford, GU2 7XH UK; 3 Department of Statistics, University of Warwick, Coventry, CV4 7AL UK

**Keywords:** Simulation-based inference, Monte Carlo, Bayesian inference

## Abstract

Approximate Bayesian computation (ABC) is a well-established family of Monte Carlo methods for performing approximate Bayesian inference in the case where an “implicit” model is used for the data: when the data model can be simulated, but the likelihood cannot easily be pointwise evaluated. A fundamental property of standard ABC approaches is that the number of Monte Carlo points required to achieve a given accuracy scales exponentially with the dimension of the data. Prangle et al. (2018) proposes a Markov chain Monte Carlo (MCMC) method that uses a rare event sequential Monte Carlo (SMC) approach to estimating the ABC likelihood that avoids this exponential scaling, and thus allows ABC to be used on higher dimensional data. This paper builds on the work of Prangle et al. (2018) by using the rare event SMC approach within an SMC algorithm, instead of within an MCMC algorithm. The new method has a similar structure to SMC$^{2}$ (Chopin et al. 2013), and requires less tuning than the MCMC approach. We demonstrate the new approach, compared to existing ABC-SMC methods, on a toy example and on a duplication-divergence random graph model used for modelling protein interaction networks.

## Introduction

### Approximate Bayesian computation

This paper concerns Bayesian inference of the parameters θ of “implicit” models: those where the likelihood cannot be evaluated pointwise at θ. This problem is encountered in a number of different fields, including epidemiology, ecology, economics, particle physics, cosmology and genetics. Suppose we wish to use a model $f_{\theta }\left( y\right)$ for observed data *y* that depends on parameters θ, and wish to estimate the parameters of the model using Bayesian inference.

Our aim in performing inference depends on the problem we are tackling. Three possible aims are:

- *Inference* of the parameters θ or functions of θ, e.g. the reproduction number $R_{0}$ in an epidemic;
- *Prediction* of possible future data using the inferred model, e.g. number of new infections in an epidemic;
- *Model criticism or comparison* to help us understand if *f* is a realistic model, either in terms of prediction, or in terms of describing the observed data.

In each situation the posterior distribution $\pi \left( \theta \mid y\right)$ of parameters θ plays a central role. For many commonly used choices of *f*, we can evaluate $f_{\theta }\left( y\right)$ pointwise at θ. Inference then usually proceeds using a Monte Carlo method for simulating from the posterior distribution $\pi \left( \theta \mid y\right) \propto p\left( \theta \right) f_{\theta }\left( y\right)$, for example importance sampling (IS), Markov chain Monte Carlo (MCMC) or sequential Monte Carlo (SMC). The standard versions of these algorithms all rely on being able to evaluate the prior and likelihood pointwise at θ, thus cannot be directly implemented for implicit models. This led to the development of approximate Bayesian computation (ABC), which replaces the likelihood with an assessment as to whether θ is plausible under the posterior by simulating $x\sim f_{\theta }\left( \cdot \right)$ and checking if statistics of *x* are close to statistics of the observed data *y*. This procedure provides an approximation to the likelihood (the “estimated ABC likelihood”), the use of which results in an approximation to the posterior (“ABC posterior”).

ABC is a well-established area of research. Much work has focussed on two issues: how to explore the space of θ whilst using the estimated ABC likelihood; and the effect of using the ABC approximation in place of the exact likelihood. Methods for exploring the space of θ include the ABC variants of rejection sampling, IS, MCMC, SMC and Bayesian optimisation: a review can be found in Fan and Sisson (2019). The development of methods for improving the estimation of the likelihood has focussed on improving both the bias and variance of the estimates. We continue our introduction by introducing the ABC-MCMC algorithm, and discussing the bias and variance of the estimated ABC likelihood in this context.

### ABC-MCMC and the estimated ABC likelihood

Let $P_{\epsilon }\left( y\mid x\right)$ be the “ABC kernel”: a distribution on y∣x, symmetric in *x*, that is specified by normalising a kernel with tolerance (or bandwidth) $\epsilon \ge 0$, which takes larger values the closer *x* is to *y*. The ABC-MCMC algorithm of Marjoram et al. (2003) is shown in Algorithm 1.

**Figure:**
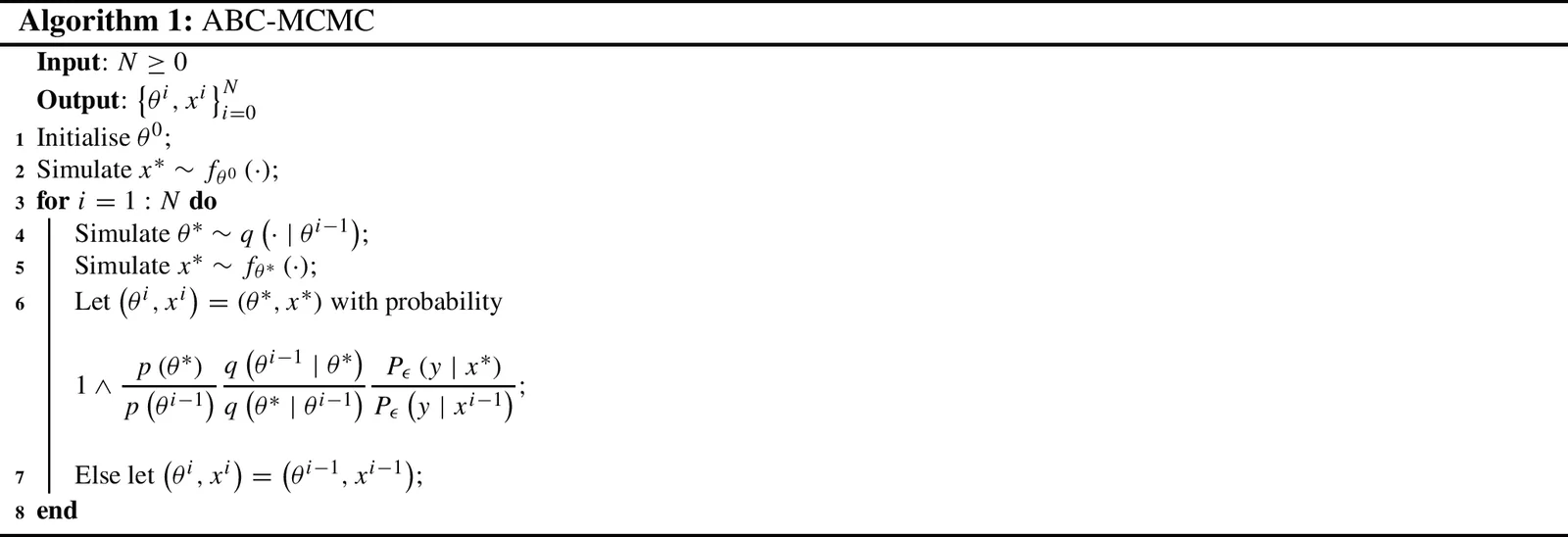

One way of understanding ABC-MCMC is as a “pseudo-marginal” method (Beaumont 2003; Andrieu and Roberts 2009). This class of methods uses an unbiased approximation to the likelihood in place of an exact likelihood in a Metropolis-Hastings algorithm; it is shown in Andrieu and Roberts (2009) that the limiting distribution of the θ-points generated by such an algorithm is the same as if the exact likelihood were used. In the case of ABC–MCMC, the likelihood estimate at a point θ is simply $P_{\epsilon }\left( y\mid x\right)$, where $x\sim f_{\theta }\left( \cdot \right)$. This is a Monte Carlo estimate of what we call the “true” ABC likelihood

$$ l \left( y\mid \theta \right) =\int _{x}P_{\epsilon }\left( y\mid x\right) f_{\theta }\left( x\right) dx. $$

This integral is intractable in general, hence the use of a Monte Carlo estimator. As pointed out in Del Moral et al. (2012), the estimated ABC likelihood $P_{\epsilon }\left( y\mid x\right)$ is a very high variance estimate of the true ABC likelihood since it uses only a single Monte Carlo point, and in some circumstances it may be more efficient to take the sample average of $P_{\epsilon }\left( y\mid \cdot \right)$ for several simulations from $f_{\theta }\left( \cdot \right)$. In this case, for $N_{x}$ points simulated from $f_{\theta }\left( \cdot \right)$, the estimated likelihood is

1 $$\begin{aligned} \hat{l}\left( y\mid \theta \right) =\frac{1}{N_{x}}\sum _{n=1}^{N_{x}}P_{\epsilon }\left( y\mid x^{n}\right) . \end{aligned}$$

For expository purposes, it is useful to view our Monte Carlo estimator as an IS estimator of the normalising constant $\int _{x}P_{\epsilon }\left( y\mid x\right) f_{\theta }\left( x\right) dx$ of the unnormalised target distribution $P_{\epsilon }\left( y\mid x\right) f_{\theta }\left( x\right)$ when using proposal $f_{\theta }\left( x\right)$. The importance sampling estimator is unbiased, and its variance of an depends on the distance between the proposal and the target (Agapiou et al. 2017). Andrieu and Roberts (2009) tells us that the unbiasedness of the estimated likelihood will result in ABC-MCMC having the same invariant distribution as if we had used the true ABC likelihood.

A likelihood estimator with a higher variance usually results in a less efficient MCMC algorithm. For the estimated ABC likelihood, we have that the distance between target $P_{\epsilon }\left( y\mid x\right) f_{\theta }\left( x\right)$ and proposal $f_{\theta }\left( x\right)$ (and hence the variance of the estimator), will tend to be larger when the dimension of *y* is higher and when ϵ is smaller. The dimension of *x* has a particularly large impact: the variance of the estimator increases exponentially with the dimension of *y*. This is the reason that the raw data is rarely used in ABC: common practice is to reduce the dimension of the data by using summary statistics, and thus reduce the variance of the likelihood estimator. If the statistics are not sufficient, this results in a different posterior to using the full data: the variance has been reduced at the cost of introducing bias. A similar tradeoff is made when choosing an appropriate ϵ. The ABC likelihood will only result in a posterior equal to the true posterior as $\epsilon \rightarrow 0$, however this is the case where the variance of the likelihood estimator is at its highest. In practice some ϵ>0 is used, chosen such that the variance of estimates from ABC-MCMC are not too high, although at the cost of introducing a bias.

The methodology used in this paper is focussed on trying to avoid, as far as possible, the need to reduce the dimension of the data by choosing summary statistics, whilst avoiding a high variance likelihood estimator. The approach we use is described in the following section.

## Rare event ABC-SMC

### Reparameterisation trick

Many methods for estimating the likelihood can be seen as different ways of approaching this bias-variance tradeoff: for example some approaches assume some parametric or non-parametric model for joint or conditional distributions of θ,y, with the aim of reducing variance whilst attempting to introduce little additional bias (Cranmer et al. 2020). Our method is one in an alternative class of approaches that make use of a decomposition of the simulator: rewriting the simulator as a deterministic transformation *H* of the parameter θ and a random vector *u* that is drawn from a tractable distribution $\phi \left( \cdot \mid \theta \right)$. We believe this “reparameterisation trick” was first introduced in the ABC context in Andrieu et al. (2012); it has been used in a number of papers since (e.g. Meeds and Welling (2015); Forneron and Ng (2016); Moreno et al. (2016); Graham and Storkey (2017)). The important property of this decomposition is that it allows us to replace the internal target

$$ P_{\epsilon }\left( y\mid x\right) f_{\theta }\left( x\right) $$

with

$$ P_{\epsilon }\left( y\mid H\left( u,\theta \right) \right) \phi \left( u \mid \theta \right) . $$

Although we are still using an ABC-style likelihood, the intractable $f_{\theta }$ is no longer present. This presents the possibility of exploring other ways of moving around the $\left( \theta ,u\right)$-space, for example the Hamiltonian Monte Carlo (HMC) in Graham and Storkey (2017)). To understand the possible benefits of using this approach, it is useful to think again about the reasons for the high variance of the standard ABC likelihood: in this case the *u* variable is drawn independently at each iteration, from a distribution that does not depend on *y*. We might hope that by tailoring *u* specific to θ and *y* - choosing the random vector *u* such that our likelihood simulations conditional on θ are close to *y* - we make an efficiency gain.

### Rare event SMC

In this paper we follow Prangle et al. (2018) in using a "rare event" SMC algorithm for simulating from the conditional distribution of $u\mid \theta ,y$ in order to tailor *u* to θ and *y*. In fact, this simulation of *u* conditional on *y* and θ is used to estimate the ABC likelihood $l^{\text {}}\left( y\mid \theta \right) =\int _{u}P_{\epsilon }\left( y\mid H\left( u,\theta \right) \right) \phi \left( u \mid \theta \right) du$, with a lower variance than the standard approach, which we presented above as importance sampling. This is exactly the marginal particle MCMC algorithm (Andrieu et al. 2010) in the case of ABC. Theoretical results about SMC tell us that we expect the variance of this likelihood estimator to scale more favourably with the dimension of *y* (which for simplicity we assume is the same as the dimension of *u*) than the standard IS based estimator. Prangle et al. (2018) studies theoretically the computation required to accept a proposed point using the IS estimator compared to the SMC as $d\rightarrow \infty$, and finds a cost of $O\left( \epsilon ^{-d} \right)$ for the IS approach, compared to $O\left( d^2 \log \left( \epsilon ^{-1} \right) \right)$ for SMC (also see Beskos et al. (2014)).

**Fig. 1:**
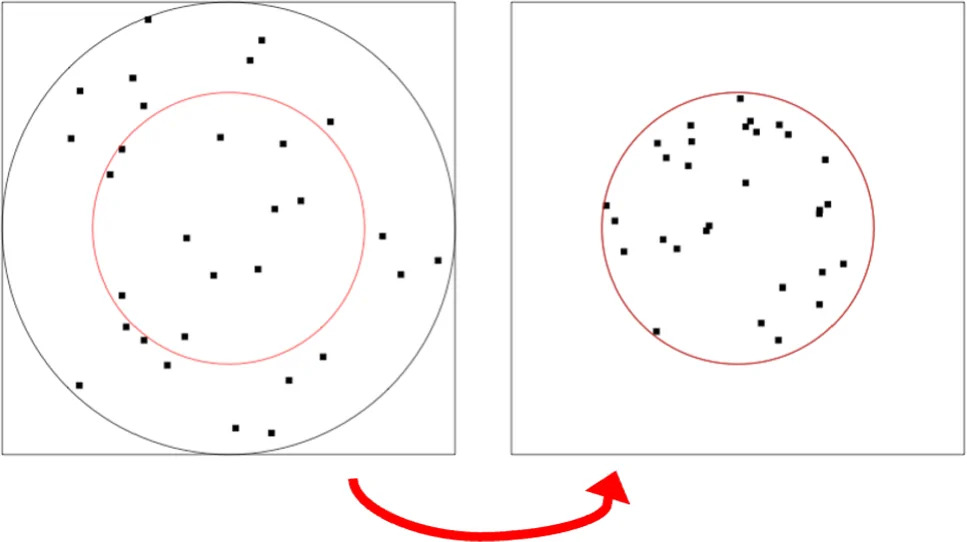
Illustration of the inner step of a rare event ABC-MCMC algorithm for a single point proposed in θ-space. The black dots represent draws from the model (*u*-points transformed by *H* so that they are in the data space). A uniform ABC kernel is used, so that the circles represent regions of non-zero density for this kernel. The outer circle in the left panel represents the ABC kernel for tolerance $\epsilon _{t-1}$, with the black dots in the left panel representing the $N_{u}$ points from the $\left( t-1\right)$th step of the algorithm. The right panel shows the same points after reweighting, resampling and an MCMC move

This approach should not be confused with ABC-SMC algorithms, which are described later in this paper. These algorithms are focussed on using SMC to explore θ-space (sometimes based on a justification of exploring the joint $\left( \theta ,u\right)$-space). The SMC method employed by Prangle et al. (2018) is instead exploring the conditional distribution of $u\mid \theta ,y$. When the ABC kernel is a uniform distribution, such an SMC algorithm has been explored thoroughly in the work of Cérou et al. (2012), under the guise of a method for rare event estimation. For the situation of ABC, we use a sequence of *T* internal targets with the final one being $P_{\epsilon }\left( y\mid H\left( u,\theta \right) \right) \phi \left( u \mid \theta \right)$. The “0th internal target” (the proposal) is given by $\phi \left( u \mid \theta \right)$, and the *t*th internal target (for $1\le t\le T$ is $P_{\epsilon _{t}}\left( y\mid H\left( u,\theta \right) \right) \phi \left( u \mid \theta \right)$, where $\infty>\epsilon _{1}>...>\epsilon _{T}=\epsilon$. The algorithm then proceeds as in Algorithm 2; our notation is that the values taken by particles in the SMC sampler have a $\left( \cdot \right) {}^{n}$ superscript; so for example $u_{t}^{n}$ is the *u*-value taken by the *n*th particle at the *t*th internal target. $\mathcal {M}\left( \left( w_t^1,..., w_t^{N_u} \right) \right)$ in the resampling step stands for the multinomial distribution which assigns probability $w_t^n$ to outcome $n \in 1:N_u$. The algorithm mentions that resampling will be performed if some degeneracy condition is met: the most common choice is if the effective sample size, estimated using

$$ \left( \sum _{n=1}^{N_{\theta }} \left( w^n\right) ^2 \right) ^{-1} $$

falls below some proportion $\alpha \in (0,1)$ of $N_u$. An illustration of the rare event approach can be found in Fig. 1.

**Figure:**
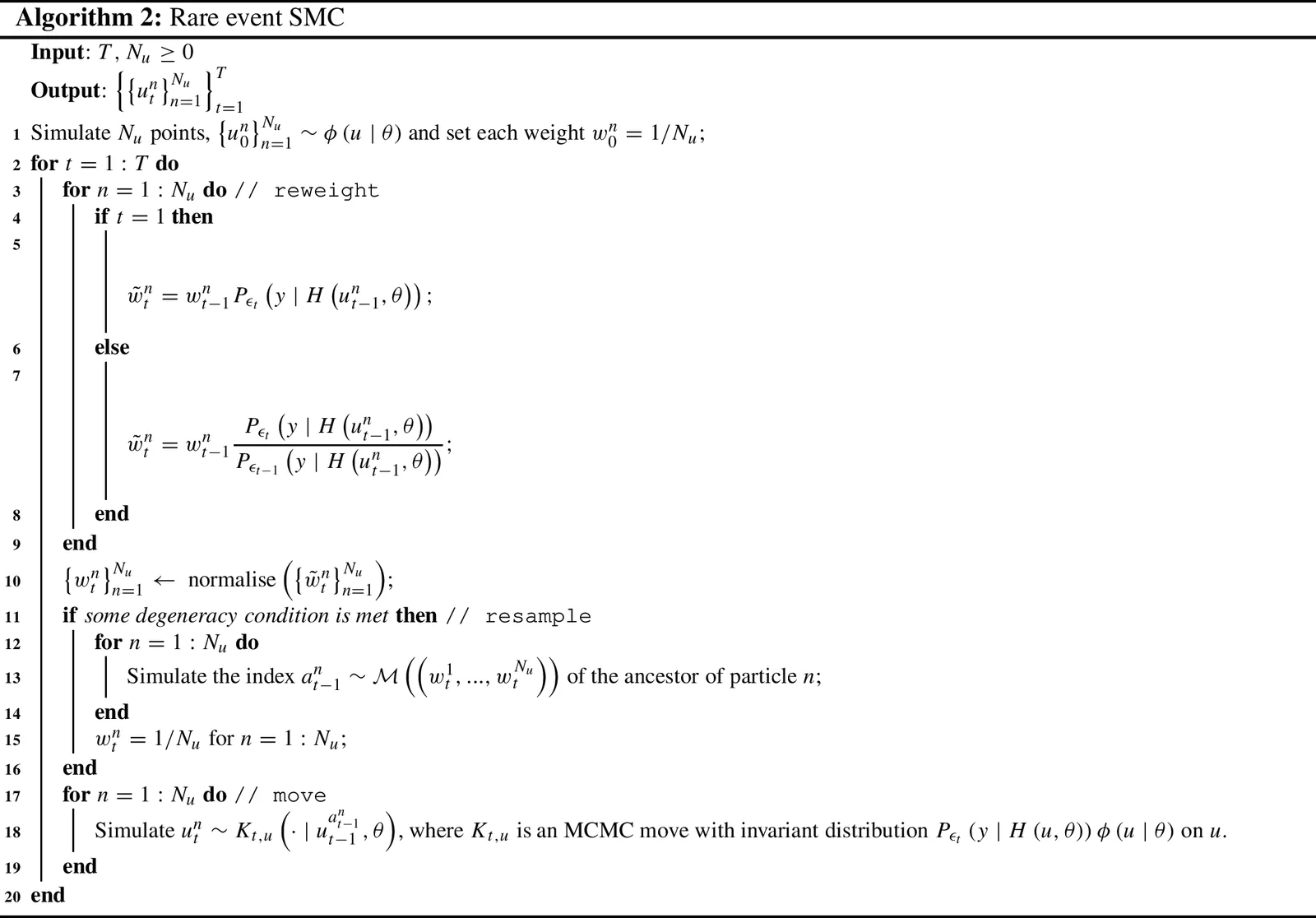

For this method to be efficient, the MCMC needs to be well-designed, accounting for the fact that $\epsilon _{t}$ is decreasing at each iteration. Prangle et al. (2018) find a slice sampler to be an efficient choice here.

After running this algorithm, the ABC likelihood can be estimated using

2 $$\begin{aligned} \overline{l}\left( y\mid \theta \right) = \prod _{t=1}^T \sum _{n=1}^{N_{u}} \tilde{w}^n_{t}. \end{aligned}$$

### Overview of the rest of the paper

This paper introduces a new approach to using ABC when *y* is high-dimensional, through combining ABC-SMC for exploring θ-space with rare event SMC for estimating the ABC likelihood. The aim is to gain the strengths of both approaches.

- *Rare event ABC-MCMC* scales better than standard ABC-MCMC as the dimension of the data grows. However, both methods are highly dependent on tuning - primarily, how a user should choose ϵ to tradeoff bias and variance expense in a sensible way. In addition, neither method can be used to estimate the model evidence.
- *ABC-SMC* allows ϵ to be chosen adaptively, and provides an estimate of the model evidence. Also, it uses a population to explore θ-space, allowing the move step to be tuned as the algorithm runs. However, it uses the standard ABC likelihood estimate (equation (1)), which leads to poor performance for high-dimensional *y*.

In Sect. 3 we introduce the new method, followed by presenting empirical results in Sect. 4 and conclusions in Sect. 5.

## Rare-event ABC-SMC$^{2}$

We begin this section by describing ABC-SMC, before moving on to the new approach.

### ABC-SMC

The use of SMC samplers in the ABC setting was pioneered by Sisson et al. (2007), with the key idea being to use as a sequence of distributions a sequence of ABC posteriors with decreasing tolerances $\epsilon _{t}$ for t=1:T, where $\epsilon _{1}>...>\epsilon _{T}$. The first tolerance $\epsilon _{1}$ is typically chosen to give an ABC posterior close to the prior $p\left( \theta \right)$, which is typically used as the initial distribution $\pi _{0}$. The final tolerance $\epsilon _{T}$ is chosen to be the desired tolerance, such as we would use in an ABC-MCMC algorithm.

**Fig. 2:**
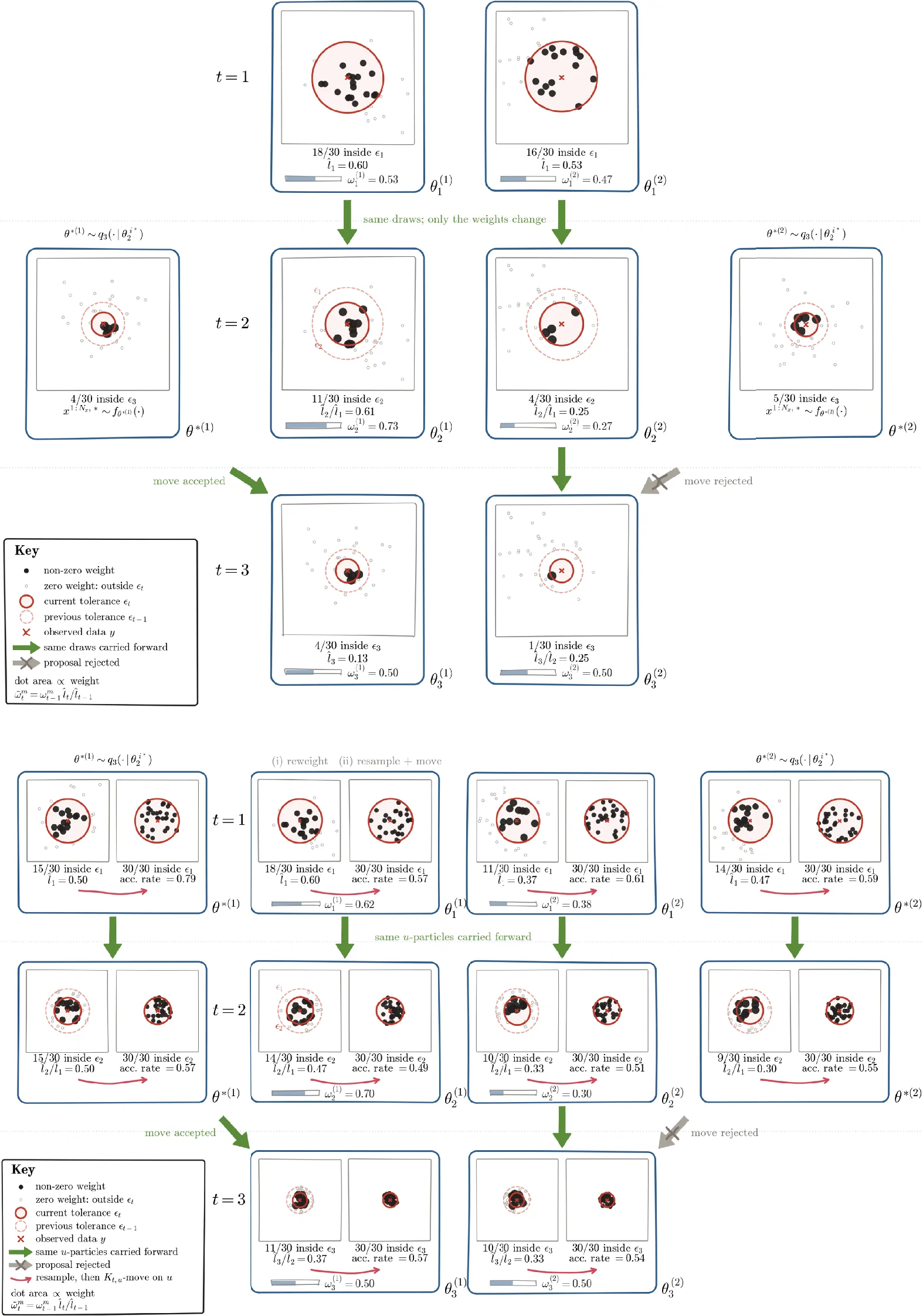
An illustration of ABC-SMC (upper) and RE-ABC-SMC$^{2}$ (lower) with two θ-particles for three iterations, including MCMC updates (with proposals on the left and right edges of the figures). Each particle has its own draws from the model, which are represented by black dots. A uniform ABC kernel is used, with the region of non-zero density for this kernel being represented by the circle. For ABC-SMC, once the draws from the model (the black/grey points) are made for each particle, they remain the same throughout the algorithm, unless a θ-particle is replaced using an MCMC move, whereas for RE-ABC-SMC$^{2}$ the points are updated using an internal MCMC

In this paper we use the variation on the idea introduced by Del Moral et al. (2012). In this method the sequence of (unnormalised) “external” targets is $P_{\epsilon _{t}}\left( y\mid x\right) f_{\theta }\left( x\right) p\left( \theta \right)$ for t=1:T, with the initial distribution being $f_{\theta }\left( x\right) p\left( \theta \right)$. In Algorithm 3 we describe the form of ABC-SMC that uses a likelihood estimate based on $N_{x}$ points drawn from the likelihood for each θ: for the *n*th one of these points drawn for the *m*th particle in θ-space, we use the notation $x_{t,\theta }^{n,m}$. Each step of the algorithm uses the likelihood estimate from Eq. (1), i.e. for any *s*, *t* we use

3 $$\begin{aligned} \hat{l}_s \left( y\mid \theta ^n_t\right) =\frac{1}{N_{x}}\sum _{n=1}^{N_{x}}P_{\epsilon _s}\left( y\mid x_t^{n,m}\right) . \end{aligned}$$

This version of the algorithm differs from the one in Del Moral et al. (2012), since we only use an MCMC move when a degeneracy condition is met. The sampling from the mixture distribution in the algorithm is one way of writing a resampling step, followed by an MCMC move. The reason for writing the algorithm in this way is to make a direct comparison with the SMC$^{2}$ algorithm of Chopin et al. (2013), which we build on in the next section. A figure illustrating the algorithm is shown in the upper panel of Fig. 2 and a video illustration the steps of the algorithm can be found here.

**Figure:**
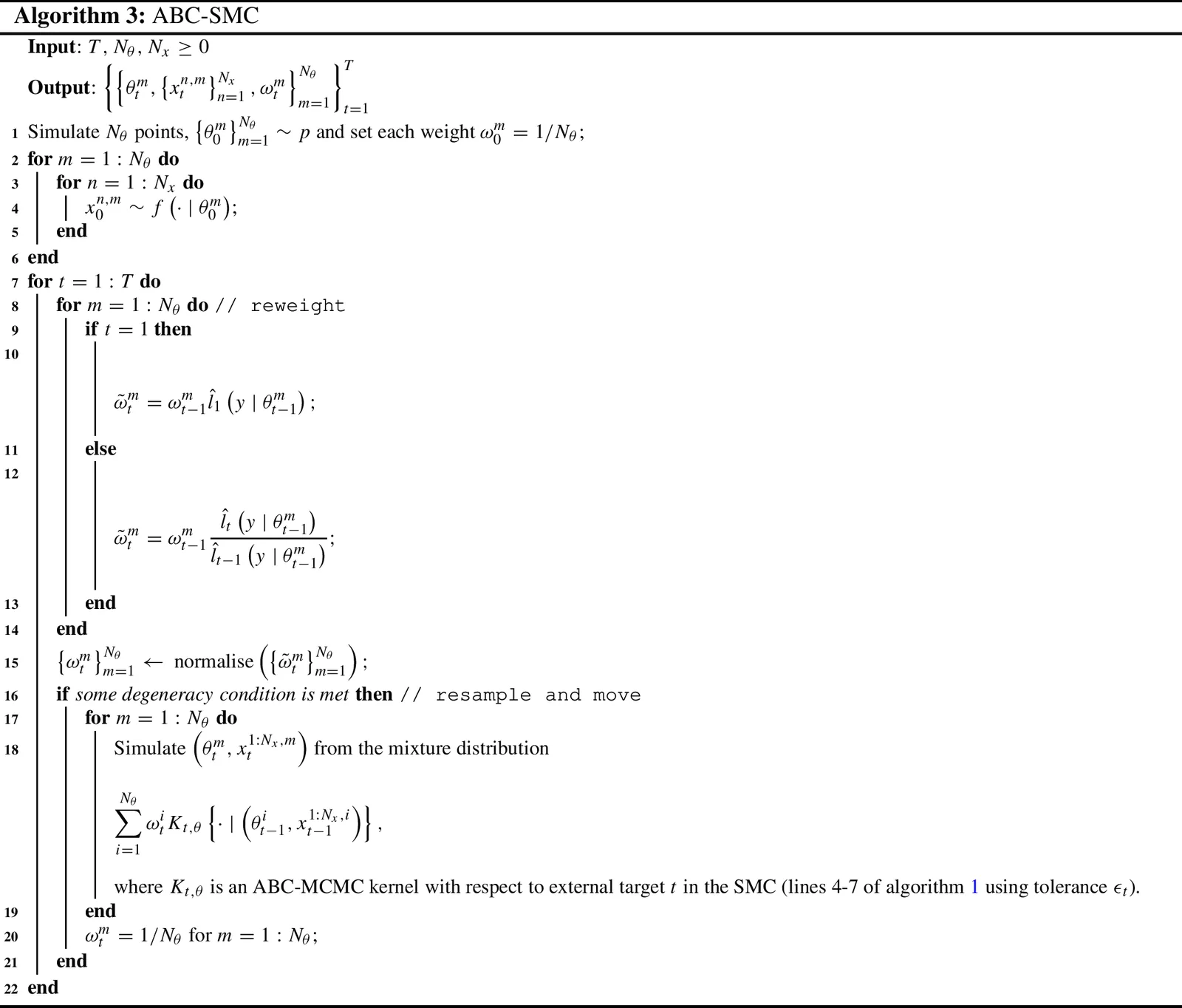

### Rare-event ABC-SMC$^{2}$

#### Algorithm

In this section, we introduce our new approach: the use of the structure of the SMC$^{2}$ algorithm of Chopin et al. (2013) in the ABC setting, through using rare event ABC to estimate likelihood ratios when required. SMC$^{2}$ is designed for a state space model setting: $y_{1:t}$ are noisy observations of a latent time series $x_{1:t}$. The generative model for this situation is specified in two parts: $f_{\theta }\left( x_{1:t}\right)$, which models the dynamics of the latent time series, and $g_{\theta }\left( y_{1:t}|x_{1:t}\right)$. which models the distribution of the observations. SMC$^{2}$ may be used to estimate the posterior distribution on both θ and $x_{1:t}$. It is set up using an “external” SMC on θ-space, and an “internal” SMC on *x*-space conditional on θ. Each iteration of the external SMC algorithm necessitates running an additional step of a separate internal SMC algorithm for each particle in the external SMC.

The internal SMC has internal target $f_{\theta }\left( x_{1:t}\right) g_{\theta }\left( y_{1:t}|x_{1:t}\right)$ at iteration *t*. The weights calculated for each internal particle when updating the internal SMC at iteration t+1 can be used to estimate $p\left( y_{t+1}\mid y_{1:t},\theta \right) =p\left( y_{1:t+1}\mid \theta \right) /p\left( y_{1:t}\mid \theta \right)$ for each external particle, this being the term needed in the weight update in the external SMC. Justification that the algorithm has the desired target distribution can be found in section A.

For our description of the algorithm, we follow as closely as possible, the notation in Chopin et al. (2013). The “internal” SMC algorithm is the rare event SMC method introduced in section 3.1. The “external” SMC algorithm is given in Algorithm 4. A figure illustrating the algorithm is shown in the lower half of Fig. 2 and a video illustration the steps of the algorithm can be found here.

**Figure:**
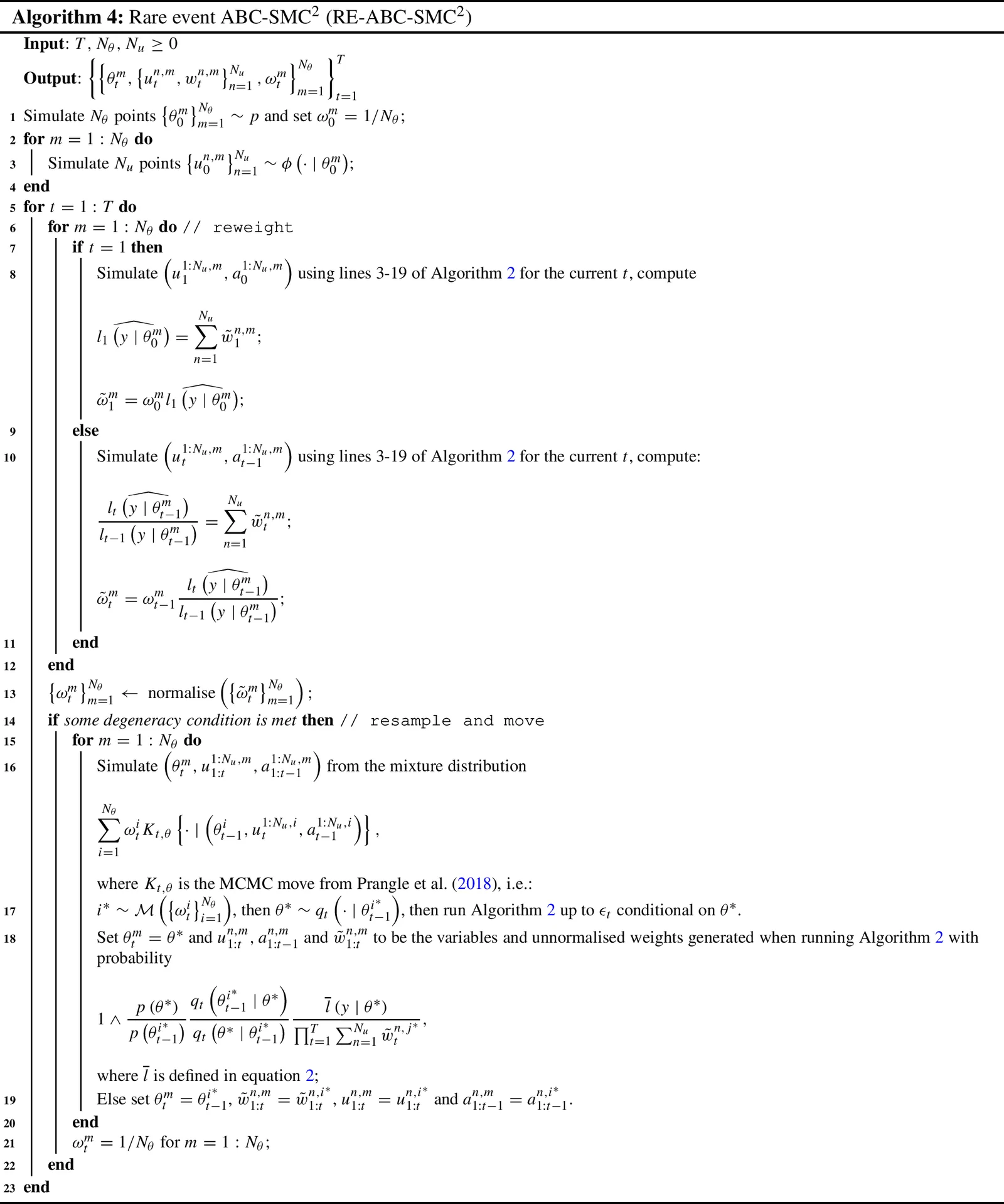

#### Scaling with data dimension

Beskos et al. (2014) gives the result that to control the variance of estimators from an SMC algorithm on a space of dimension *d*, *O*(*d*) intermediate distributions are required. Together with the evaluation of a *d*-dimensional target at each time step, this gives a total cost of $O(Nd^2)$, where *N* is the number of particles.

For RE-ABC-SMC$^2$, the computational cost is dominated by the estimation of the likelihood when performing an MCMC move on θ-space. Therefore the total cost of the algorithm is given by considering the cost of each move, and the number of moves that are performed:

- *Cost of each move*. Each move requires running an SMC sampler on *u*-space and controlling the variance of the likelihood estimator, which using (Beskos et al. 2014), has a cost of $O(N_{u}d^2)$, where *d* is the dimension of the data *y*.
- *Number of moves*. We perform moves for each θ-particle so, supposing the move step is employed at each iteration, the number of moves is $N_{\theta } T$, where *T* is number of distributions used in the outer SMC. *T* is chosen adaptively so as to control the variance of the internal likelihood estimators, so has cost *O*(*d*).

Using this heuristic argument, the complexity of RE-ABC-SMC$^2$ is $O(N_{\theta } N_{u}d^3)$. This renders the algorithm computationally expensive as $d_{y}$ grows, but is preferable to the corresponding $O(N_{\theta } N_{x} \exp (d))$ of ABC-SMC.

### Implementation details

Algorithm 2 may be modified in several ways in order to, at each iteration, make use of the current set of weighted particles to inform subsequent steps of the algorithm. These modifications allow many of the tuning parameters of the algorithm to be chosen adaptively. This section describes the adaptive approaches used in this paper.

#### Adapting the sequence of tolerances

One of the appealing properties of ABC-SMC (Algorithm 3) in comparison with ABC-MCMC (Algorithm 1) is that the choice of the tolerance ϵ may be automated in ABC-SMC. The most commonly-used approach is that of Del Moral et al. (2012). This method adds an additional routine before line 8 of Algorithm 3 to determine the choice of $\epsilon _t$ to be used in the current iteration of the SMC. We make use of the fact that $\hat{l}_{t}\left( y\mid \theta _{t-1}^{m}\right)$ can be computed very cheaply for different values of ϵ given that $\left\{ x_t^{n,m} \right\} _{n=1}^{N_x}$ have already been simulated. This means that the weights $\left\{ \tilde{\omega }^m_{t} \right\} _{m=1}^{N_{\theta }}$ may be calculated for many different choices of ϵ, with the most appropriate value of ϵ chosen. Usually a bisection algorithm is used to search for the most appropriate ϵ within the range $[0,\epsilon _{t-1}]$. The most appropriate ϵ is usually chosen by finding the ϵ that results in the estimated conditional effective sample size (CESS) (Zhou et al. 2015) being closest to some proportion $\beta \in (0,1)$ of $N_{\theta }$. The CESS, defined by

$$ {CESS}_{t}=\frac{N_{\theta } \left( \sum _{m=1}^{N_{\theta }} \omega _{t-1}^{m} \frac{\hat{l}_{t}\left( y\mid \theta _{t-1}^{m}\right) }{\hat{l}_{t-1}\left( y\mid \theta _{t-1}^{m}\right) }\right) ^{2}}{\sum _{m=1}^{N_{\theta }} \omega _{t-1}^{m}\left( \frac{\hat{l}_{t}\left( y\mid \theta _{t-1}^{m}\right) }{\hat{l}_{t-1}\left( y\mid \theta _{t-1}^{m}\right) }\right) ^{2}}, $$

was introduced in Zhou et al. (2015) as more appropriate measure of degeneracy between two successive steps of the algorithm than the ESS in the case when resampling is not performed at every step. This approach ensures that the sequence of distributions is not chosen to change so quickly that the SMC becomes degenerate.

A similar approach may be used in Algorithm 2. At the same stage of the algorithm (before line 6 in Algorithm 2), a bisection routine may be added to find $\epsilon _{t}$ for the next step of the algorithm. Again the CESS may be used to chose the most appropriate $\epsilon _{t}$, in this case given by

$$ {CESS}_{t}=\frac{N_{\theta } \left( \sum _{m=1}^{N_{\theta }} \omega _{t-1}^{m} \widehat{\frac{l_{t}\left( y \mid \theta _{t-1}^{m}\right) }{l_{t-1}\left( y \mid \theta _{t-1}^{m}\right) } }\right) ^{2}}{\sum _{m=1}^{N_{\theta }} \omega _{t-1}^{m}\left( \widehat{\frac{l_{t}\left( y \mid \theta _{t-1}^{m}\right) }{l_{t-1}\left( y \mid \theta _{t-1}^{m}\right) } }\right) ^{2}}, $$

Once more the calculation of this criterion is computationally cheap given the variables $\left\{ u^{n,m}_{1:t} \right\} _{n=1}^{\theta _u}$ generated at the previous step of the algorithm. In this case, to calculate the ratio $\widehat{\frac{l_{t}\left( y \mid \theta _{t-1}^{m}\right) }{l_{t-1}\left( y \mid \theta _{t-1}^{m}\right) } }$ for each candidate ϵ, we need only to run lines 3-9 of Algorithm 2.

#### Adapting the MCMC proposals

Algorithm 4 makes use of two MCMC steps: one in the external SMC, which we may think of as a move on θ, and the other in the internal SMC, which is a move on *u*.

For the MCMC move in the externaL SMC, we adopt the widely-used approach in SMC of using, at iteration *t*, a Gaussian random walk proposal in Metropolis-Hastings, with covariance $2.38^2 \hat{\Sigma _t}/d$, where $\Sigma _t$ is the (weighted) sample covariance of the current particle set before resampling.

Prangle et al. (2018) noted the importance of the internal MCMC move adapting to match the changing scale of its target as $\epsilon _t$ changes, and the same is true of the external MCMC move. Prangle et al. (2018) used a slice sampler to achieve this effect, but other approaches are also possible, such as using the current population of particles to estimate the scale of a proposal for a Metropolis-Hastings algorithm, as is used for example in Everitt and Rowińska (2021). In Sect. 4 we adopt approaches tailored to each application.

#### Adapting the number of MCMC moves on θ

As ABC-SMC, Algorithm 3 runs, $\epsilon _t$ decreases, and it becomes more unlikely that the simulation *x* is close to *y*. The result is that the acceptance rate of the ABC-MCMC moves used by the algorithm decreases as *t* increases. Eventually the acceptance rate drops to zero, and whilst the tolerance $\epsilon _t$ continues to decrease and thus resampling is performed, the population ends up consisting of many duplicated particles. We observed a similar effect when running RE-ABC-SMC$^2$, but for a much lower tolerance than for ABC-SMC.

One approach to avoiding this issue is to adapt the number of MCMC iterations used as the algorithm progresses. South et al. (2019) suggest the following approach to choosing the number of iterations for which to run a Metropolis-Hastings algorithm: at each SMC iteration they examine the acceptance rate (denoted $\hat{p}_{\text{ acc }}$) across all particles of the first iteration of the MCMC move, and choose the number of iterations to be

$$ \Bigg \lceil \frac{\log (c)}{\log \left( 1-\hat{p}_{\textrm{acc}}\right) }\Bigg \rceil , $$

with $\big \lceil \cdot \big \rceil$ denoting the ceiling function, such that there is an estimated probability of 1-c that each particle is moved at least once.

We use this adaptive approach in each implementation of ABC-SMC or RE-ABC-SMC$^2$, but also impose a cap on the number of moves.

#### Tuning the rare event likelihood estimator

The efficiency of the rare event ABC-MCMC move is fundamental to the performance of the algorithm, and it has two parameters that must be tuned: the number of MCMC moves on *u* at each iteration and the number of particles $N_u$. To tune the number of MCMC moves, the adaptive approach from Sect. 3.3.3 can be used: each subsection in 4 describes the specific approach we chose in each application.

The results of Sherlock et al. (2015); Doucet et al. (2015) provide guidance as to how to tune $N_u$ so as to ensure the efficiency of the rare event ABC-MCMC move: they recommend choosing $N_u$ so that the variance of the log-likelihood estimator is 1-3. The situation is complicated in our algorithm by the fact that the reducing tolerance will lead to an increase in the variance of the estimated likelihood across SMC steps. A conservative approach to choosing $N_u$ would be to first determine the lowest tolerance to be used in the SMC (possibly through a pilot run), then to choose $N_u$ suitably for this target following (Sherlock et al. 2015; Doucet et al. 2015) (or even the more robust approach in (Sherlock 2024)). In practice we found that better performance (per computational cost) was attained by choosing a smaller $N_u$ than in these papers, and in Sect. 4 we chose $N_u$ such that the variance of the log-likelihood estimate was around 10-15. The reduction in tolerance as the algorithm progresses suggests choosing $N_u$ adaptively, but for simplicity we did not consider this in the paper.

#### Splitting different sources of randomness

Section 2 describes how we make use of the reparameterisation of a simulator model as a deterministic transformation $H\left( u,\theta \right)$ of the parameter θ and the random vector *u*, where *u* encompasses all of the stochasticity in the model. We then use MCMC moves on *u* within the algorithm. However, for some models, such as that in Sect. 4.4, the space of *u* can be extremely complicated: e.g. it may be of variable dimension. Whilst in principle it is possible to design MCMC moves for such spaces, in practice it may not be possible to do so effectively. Therefore it may be desirable to split *u* in two parts: $u_s$, which will be updated by the internal MCMC, and $u_r$ which will not be moved by the internal MCMC.

Since $u_r$ remains fixed, the MCMC move on *u* is not irreducible. However this does not prevent Algorithm 4 being a valid SMC sampler. In fact, when $u=u_r$ (i.e. none of the *U* variables are updated in the internal MCMC), we see that the original ABC-SMC method, Algorithm 3, is recovered as a special case of the new method in Algorithm 4.

## Empirical results

This section contains results for four different applications. Section 4.1 studies a toy Gaussian model to explore the properties of the method on an example for which we know the ground truth; Sect. 4.2 illustrates the use of the new approach for model comparison on moving average models; 4.3 compares the performance of the method with standard ABC on the Lotka-Volterra model where the posterior takes on a variety of shapes for some different values of the tolerance; and Sect. 4.4 examines the situation described in 3.3.5 where the sources of randomness are split.

### Gaussian model

We consider a slightly amended version of a Gaussian model studied in Prangle et al. (2018). The observed data consists of *d* points $y = \left\{ y_i \right\} _{i=1}^d$ simulated from a truncated univariate Gaussian distribution with μ=0, σ=3, lower bound a=0 and no upper bound. The aim of the inference is to estimate the posterior distribution of $\theta = \sigma$ given *y*. We used the uniform prior $\theta \sim \mathcal {U}\left( 0, 10\right) P_{\epsilon }$ was chosen to be the uniform distribution $P_{\epsilon }\left( y \mid x \right) = (2\epsilon )^{-1}$ for $(y-x)^2 \le \epsilon$ and 0 otherwise. We use $H\left( u,\theta \right) = \Vert \theta \Phi ^{-1}\left( u\right) \Vert$ for u∈[0,1], where $\Phi ^{-1}$ is the inverse Gaussian cdf. The MCMC move used to update *u* is the slice sampler from Prangle et al. (2018): pilot runs led us to choose two iterations of the slice sampler each iteration, which gave more reliable results in the high-dimensional examples.

For the move step on θ, we used a Metropolis-Hastings algorithm with a truncated (at zero) Gaussian proposal with variance chosen to be equal to the variance of the weighted particles after the reweighting step. The number of iterations of the MCMC was determined adaptively as described in Sect. 3.3.3, using c=0.5 up to a maximum of 3 moves. In all SMC algorithms we resample when the ESS drops below the proportion α=0.85 of the number of particles, and we chose the sequence of $\epsilon _t$ adaptively such that $\epsilon _t$ is as close as possible to giving a CESS of $\beta N_{\theta }$, where β=0.95.

We ran ABC-rejection, ABC-SMC and RE-ABC-SMC$^2$ on scenarios with d=25,50,100,200. As described in Sect. 2, we expect the performance of ABC-rejection and ABC-SMC to significantly deteriorate as *d* increases, whilst the performance of RE-ABC-SMC$^2$ should not deteriorate as quickly. We set up each experiment based on a pilot run of RE-ABC-SMC$^2$, which across all experiments was run with $N_{\theta }=100$. $N_u$ was chosen to be 25, 50, 75, 100 respectively for d=25,50,100,200. We terminated the pilot run when the acceptance rate of the Metropolis-Hastings moves became close to zero: this corresponded to a tolerance of ϵ=0.010,0.063,3.09,41.4 (3 s.f.) for d=25,50,100,200 respectively. We recorded the number of applications of *H* for RE-ABC-SMC$^2$ in each scenario, then ran ABC-rejection and ABC-SMC for a comparable number. For ABC-rejection, this resulted in $N_{\theta }=757000,2200000,3600000,5200000$ (3 s.f.) for d=25,50,100,200. ABC-SMC used $N_x = 1$ and $N_{\theta }=16900,48700,76700,107000$ (3 s.f.) for d=25,50,100,200. ABC-rejection used the final tolerance reached by ABC-SMC since no points were accepted for the final tolerance reached by RE-ABC-SMC$^2$. ABC-SMC was terminated early if the acceptance rate of the move step reached zero, otherwise its performance degrades since points are resampled without being moved.

A run of RE-ABC-SMC$^2$ for d=200 took around 4 h on an M1 Macbook Pro laptop, compared to 1 h for d=100. We did not consider the case of d>200 for reasons of computational cost.

**Fig. 3:**
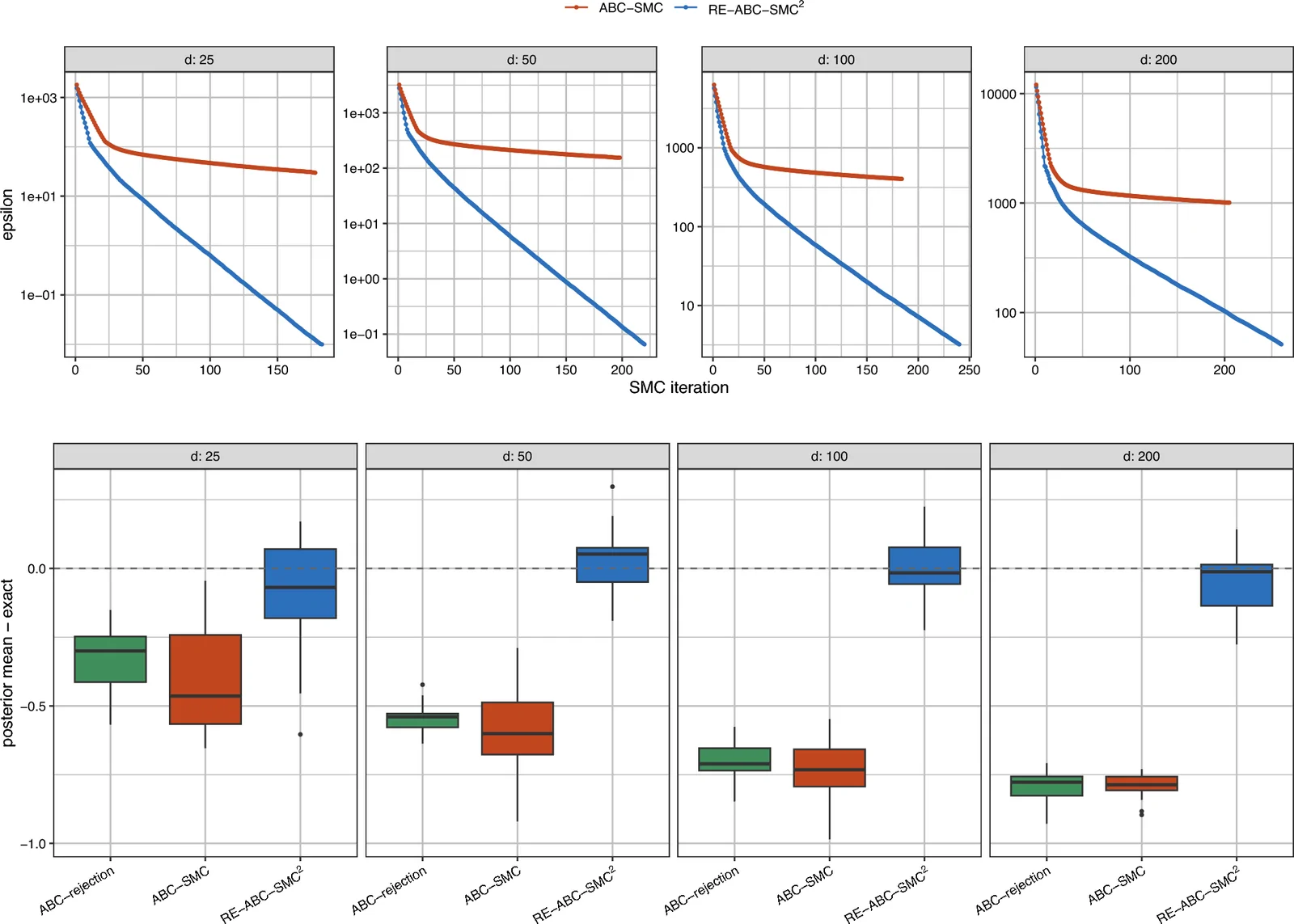
For the Gaussian example: a comparison of the tolerance (log-scale) used in runs of ABC-SMC and RE-ABC-SMC$^2$ from a representative run (top) and box plots of posterior mean estimators (15 reps) from ABC-rejection using the final tolerance of ABC-SMC, ABC-SMC and RE-ABC-SMC$^2$ (bottom)

Figure 3 (top) shows the evolution of $\epsilon _t$, as found using the adaptive algorithm from Sect. 3.3.1 over SMC iterations. We observe that the RE-ABC-SMC$^2$ algorithm is able to achieve a much lower tolerance than ABC-SMC, and that this tolerance decreases more quickly. Figure 3 (bottom) shows the relative reduced error of RE-ABC-SMC$^2$ as the dimension increases.

For this example ABC-rejection outperforms ABC-SMC due to the much larger value of $N_{\theta }$ used to match the computation – since θ is one-dimensional the cost of the SMC iterations outweighs the benefits in exploring θ-space. We might expect to see comparable performance from ABC-MCMC, as previously observed in Prangle et al. (2018).

### Moving average model

In this section we study the order-*q* moving average model (MA(*q*)) for time-series data of dimension *d*:

4 $$\begin{aligned} y_i = H_i(\theta _{1:q},u_{1:d+q}) \end{aligned}$$

for i=1:d, where

5 $$\begin{aligned} H_i(\theta _{1:q},u_{1:d+q}) = u_i + \sum _{k=1}^q \theta _k u_{i-k} \end{aligned}$$

and each $u_i \sim \phi (u_i) \mathcal {N}(u_i \mid 0,1)$. This model was studied previously in, for example, Marin et al. (2012). Following Marin et al. (2012) we impose an identifiability condition on the model through the use of a uniform prior on the region where the roots of the polynomial $1-\sum _{k=1}^q \theta _k u_{t-k} x^k$ are strictly outside the unit circle.

We simulated data of lengths d=30,100,200,300 for the MA(2), MA(3) and MA(4) models, for a parameter within the region of prior support, and used ABC-SMC and RE-ABC-SMC$^2$ to estimate the marginal likelihood for each of the MA(2), MA(3) and MA(4) models. For all experiments, we used the multivariate Gaussian ABC kernel $P_{\epsilon } (y\mid x) = \mathcal {MVN}(y\mid x, \epsilon ^2 \textbf{I}_{n})$ with a final tolerance of ϵ=1. ABC-SMC used $N_{\theta }=20000$ and $N_x=1$, and RE-ABC-SMC$^2$ $N_{\theta }=500$ and $N_u=40$. Both algorithms triggered the resample and move step using α=0.5 and used an adaptive sequence of distributions with parameter β=0.9. For the MCMC on θ we use the adaptive covariance approach described in Sect. 3.3.2, and adapt the number of moves as described in Sect. 3.3.3 with c=0.2.

Since *H* is linear, and since we use the Gaussian ABC kernel, the ABC likelihood is available in closed form for this example due to the conjugacy between the “likelihood” $P_{\epsilon }$ and “prior” ϕ. In our experiments we do not make direct use of the exact ABC likelihood, but we do make use of the fact that for any ϵ $u \mid \theta , y$ is Gaussian distributed to examine the effect of the internal move step used in the rare event likelihood estimator. Here an internal MCMC move is not required since *u* can be drawn exactly: we compare the results obtain when using these exact draws and when using an internal MCMC move. Since ϕ(u) is Gaussian, we are able to mitigate the effect of large *d* by using a preconditioned Crank-Nicolson (pCN) Metropolis-adjusted Langevin algorithm (MALA) move (Cotter et al. 2013) that – further details are in appendix B. This move is also applicable outside of the moving average model – we also use it in Sect. 4.3 for the Lotka-Volterra model.

Table 1 presents the model selection results, estimating the log-evidence for each data set for each model over 30 replications, focussing on the d=100 case. We observe that the high variance of estimates from ABC-SMC makes it unreliable in selecting the correct model, whereas RE-ABC-SMC$^2$ has a much lower variance and consistently selects the correct model. We further observe that whilst, as we would expect, using the exact Gaussian inner draw (which would not be available in most applications) reduces the variance over using pCN-MALA for the inner MCMC move, the pCN-MALA variant still produces reliable results.

**Fig. 4:**
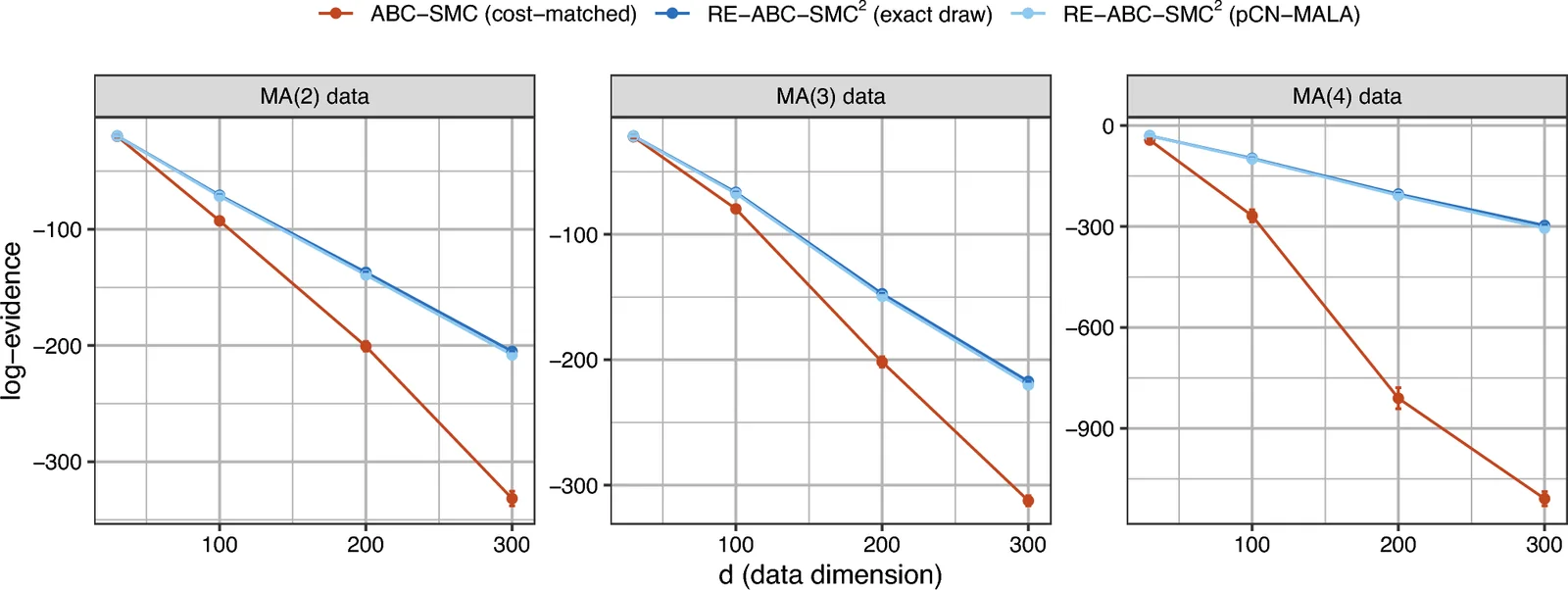
A comparison, for each moving average model fitted to the data simulated from the same model, of how the estimated log-evidence changes with the data size for each algorithm

Figure 4 illustrates the change with data length of the estimated log-evidence for each algorithm for each moving average model fitted to the data simulated from the same model. We observe that the performance of ABC-SMC is severely affected by then dimension of the data, and that the use of pCN-MALA for the inner MCMC move in the RE-ABC-SMC$^2$ produces comparatively similar results to the approach that uses exact Gaussian draws.

**Table 1:** MA model-selection log-evidence at d=100 (mean over 30 replicates, standard deviation in parentheses). Bold marks the model selected for each data-generating process (largest row evidence)

(a) ABC-SMC, cost-matched ($N_\theta$ = 20000, $N_x = 1$)			
	fitted model		
data-generating model	MA(2)	MA(3)	MA(4)
MA(2)	$\mathbf {-92.7\,(2.7)}$	-93.5(4.8)	-94.8(3.5)
MA(3)	$\mathbf {-78.9\,(2.1)}$	-79.7(2.2)	-80.7(3.6)
MA(4)	-283.2(9.7)	-272.6(11.8)	$\mathbf {-268.1\,(17.8)}$

(b) RE-ABC-SMC$^2$ (exact Gaussian inner draw)			
	fitted model		
data-generating model	MA(2)	MA(3)	MA(4)
MA(2)	$\mathbf {-70.7\,(0.2)}$	-71.6(0.3)	-72.1(0.2)
MA(3)	-67.3(0.1)	$\mathbf {-66.5\,(0.1)}$	-67.6(0.3)
MA(4)	-106.2(0.3)	-98.1(0.6)	$\mathbf {-97.5\,(0.7)}$

(c) RE-ABC-SMC$^2$ (pCN-MALA inner move)			
	fitted model		
data-generating model	MA(2)	MA(3)	MA(4)
MA(2)	$\mathbf {-71.6\,(0.3)}$	-72.4(0.3)	-73.0(0.4)
MA(3)	-68.3(0.3)	$\mathbf {-67.5\,(0.3)}$	-68.6(0.3)
MA(4)	-107.8(0.5)	-99.9(0.8)	$\mathbf {-99.5\,(1.1)}$

### Lotka-Volterra model

This section examines the performance of the new method on the stochastic Lotka-Volterra model previously studied in Papamakarios and Murray (2016); Wilkinson (2018).

We define state $x_j=(x_j^{(1)},x_j^{(2)})^\top \in \mathbb {R}_{\ge 0}^2$ consisting of prey and predator populations respectively and parameters $(\theta _1,\theta _2,\theta _3)^\top \in \mathbb {R}_{\ge 0}^3$. The chemical Langevin equation (CLE) consists of three reactions with propensity vector

$$\begin{aligned} h(x;\theta )=\begin{pmatrix}\theta _1 x^{(1)}\\ \theta _2 x^{(1)}x^{(2)}\\ \theta _3 x^{(2)}\end{pmatrix}\in \mathbb {R}^3, \end{aligned}$$

and stoichiometry matrix

$$\begin{aligned} S=\begin{pmatrix}1 & -1 & 0\\ 0 & 1 & -1\end{pmatrix}\in \mathbb {R}^{2\times 3}. \end{aligned}$$

The CLE is integrated by Euler–Maruyama with step Δt. Let *m* be the number of steps per observation interval and *d* the number of observation intervals, there are K=dm steps in total. The *u* variables in this case consists of the Gaussian noise used in each step of the solver:

6 $$\begin{aligned} u=\bigl (u_1^\top ,\dots ,u_K^\top \bigr )^\top \in \mathbb {R}^{3\times K}, \qquad u_k\sim \mathcal {N}(0,I_3), \end{aligned}$$

so that $\phi (u\mid \theta )=\mathcal {N}(0,I_{3\times K})$. This is the reparameterisation x=H(u,θ) used throughout: given *u* and θ, the map *H* defined as follows.

Each step first forms an unconstrained proposal $\tilde{x}_k$ and then applies a boundary map *b* that keeps populations finite and non-negative,

7 $$\begin{aligned} \tilde{x}_k&= x_{k-1} + S\Bigl (h_{k-1}\,\Delta t + \sqrt{h_{k-1}}\odot \sqrt{\Delta t}\,u_k\Bigr ), \nonumber \\ h_{k-1}&:=h(x_{k-1};\theta ), \end{aligned}$$

8 $$\begin{aligned} x_k&= b(\tilde{x}_k), \end{aligned}$$

where ⊙ is the elementwise product. The boundary map acts componentwise: a non-finite or overflowing entry is set to $c=10^8$, and a negative entry is reflected, x↦|x|. The observed trajectory retains only the states corresponding to observations, $H(u,\theta )=(x_0,x_m,x_{2\times m},\dots ,x_{d \times m})$; the initial condition $x_0$ is fixed.

In this example we again use the same multivariate Gaussian ABC kernel $P_{\epsilon } (y\mid x) = \mathcal {MVN}(y\mid x, \epsilon ^2 \textbf{I}_{n \times 2})$ where the data *y* consists of observations of the prey and predator sequences $y=\bigl (y_1^\top ,\dots ,y_d^\top \bigr )^\top \in \mathbb {R}^{2\times d}$. If we view the ABC kernel as part of the model (as in Wilkinson (2013)), then we observe that this model is equivalent to a state-space model where the state $x_{i \times m}$ is observed with measurement model $y \mid x \sim \mathcal {MVN}(y_i \mid x_{t \times m}, \epsilon ^2 \textbf{I}_{2} )$.

A standard method for inference in such a model is particle marginal Metropolis-Hastings (PMMH) (Andrieu et al. 2010), the results of which we use to provide us with a “ground truth” with which to compare the other algorithms, focussing on the case where we take ϵ=50. For this we used a run of 20000 iterations, discarding the first 5000 as burn in, and 300 particles in the particle filter that estimates the likelihood. The proposal was a Gaussian random walk on the log parameters with covariance $0.05^2 \textbf{I}_3$. The prior for all experiments was log uniform independently in the interval $[\exp (-6),\exp (2)]$ for each dimension.

When ABC-SMC methods are used on this example, they provide the posterior distribution for a sequence of tolerances $\epsilon _t$, with a view to estimating the posterior for a final $\epsilon _T = \epsilon$. We include this example in the paper to illustrate the ability of the new approach to estimate the posterior for each $\epsilon _t$, which in the results below we observe to have a variety of shapes.

For this example, the approach is complementary to the SMC$^2$ algorithm in (Chopin et al. 2013), where the sequence of distributions is along the sequence of observations and where ϵ is fixed or estimated as a parameter. We leave a thorough comparison with this alternative approach (which is specific to state space models) to future work.

Both ABC-SMC and RE-ABC-SMC$^2$ use SMC parameters α=0.95, β=0.85 and c=0.5 (with up to a maximum of 5 θ-space MCMC moves per SMC iteration). RE-ABC-SMC$^2$ used $N_{\theta }=1000$ and $N_u=50$. $N_{\theta }$ in ABC-SMC was chosen to be much larger at $N_{\theta }=300000$ (with $N_x=1$) to attempt to match the additional cost of RE-ABC-SMC$^2$. The internal MCMC move for RE-ABC-SMC$^2$ used pCN-MALA (appendix B) with the gradient calculated using the approach outlined in appendix C. The observed data consists of prey and predator sequences of length n=32 simulated from the model for parameters $\theta = (1,0.005,0.6)^{\top }$, where these measurements are taken every two seconds using a time step of Δt=0.05 in the solver.

**Fig. 5:**
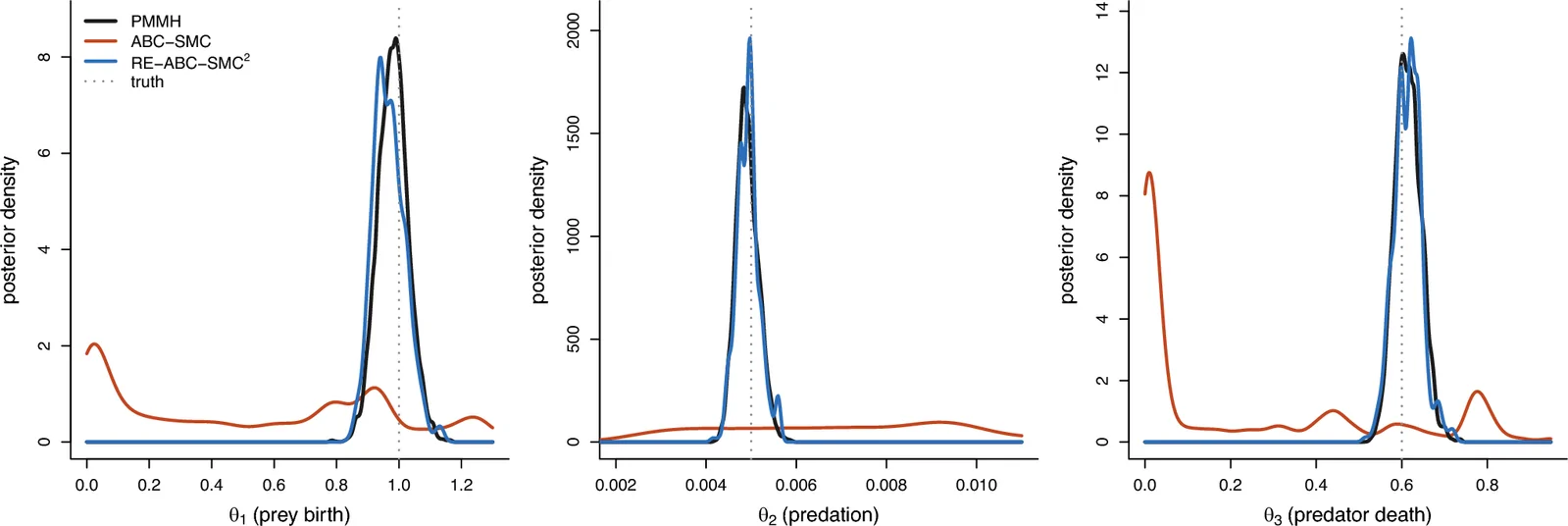
The marginal posteriors estimated by PMMH, ABC-SMC and RE-ABC-SMC$^2$

Whilst RE-ABC-SMC$^2$ reached ϵ=50, ABC-SMC only reached ϵ=236 before terminating when the acceptance rate of the MCMC move fell to zero. We chose not to further increase $N_{\theta }$ to account for this, since the results in Sect. 4.1 suggest that this would not result being able to reduce ϵ much further in ABC-SMC. Figure 5 shows the estimated posterior from ABC-SMC and RE-ABC-SMC$^2$ at the end of their runs, compared to that obtained by PMMH. We see that RE-ABC-SMC$^2$ is able to closely match the posterior from PMMH, whereas the tolerance reached by ABC-SMC is too large to allow a close match.

**Fig. 6:**
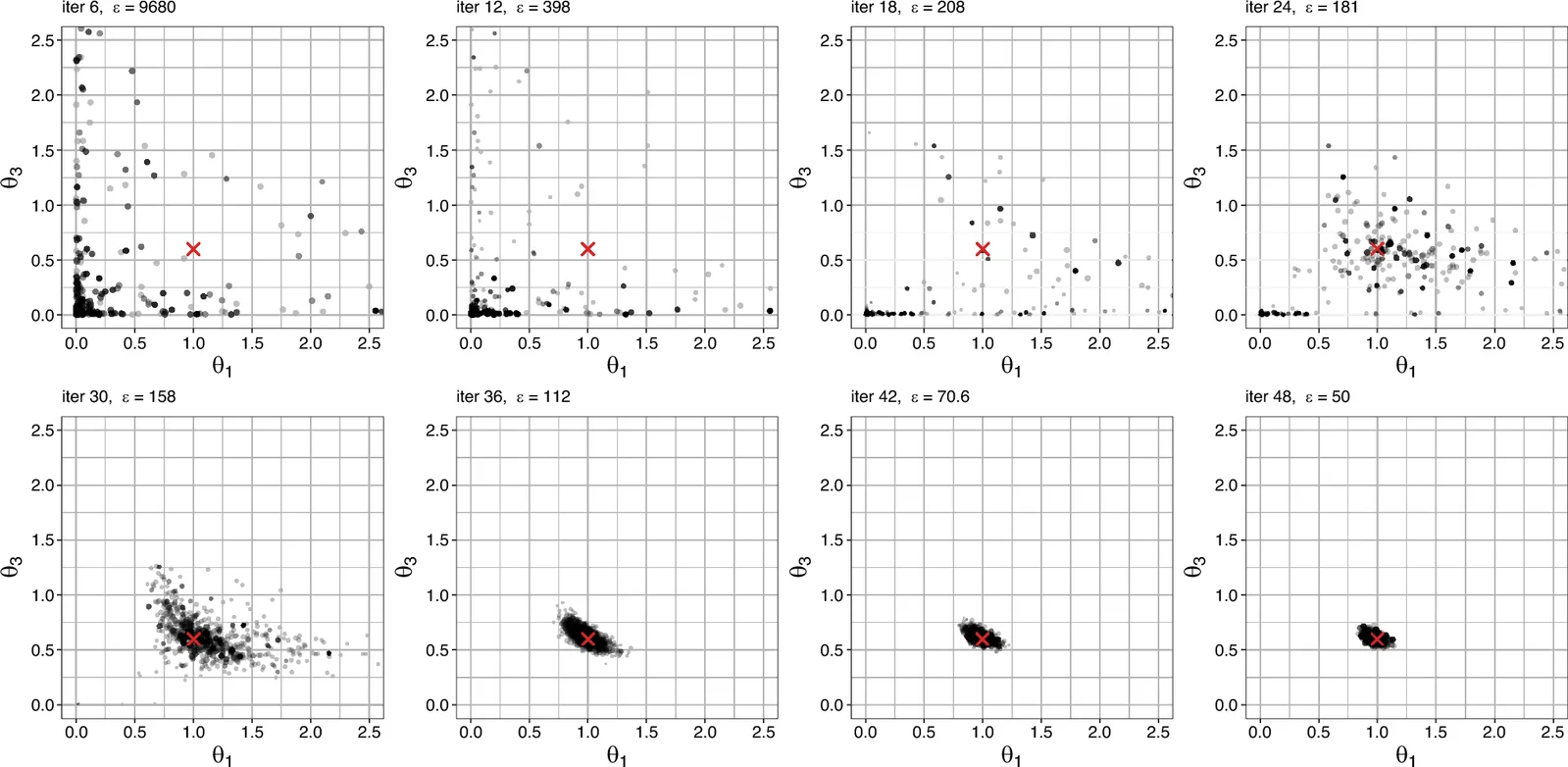
Scatter plots of the posterior on $(\theta _1,\theta _3)$ for every 6 targets in the RE-ABC-SMC$^2$

Figure 6 shows the sequence of estimated posterior distributions from RE-ABC-SMC$^2$ (plotted using the ggsmc package (Everitt 2024)), highlighting the ability of the approach to explore distributions of with different shapes across the evolution of the algorithm.

### Parameter inference in duplication-divergence random graph models

In this section we study the relative performance of ABC-SNC and RE-ABC-SMC$^2$ on a network model previously studied in Thorne and Stumpf (2012). We see in this example the use of RE-ABC-SMC$^2$ in a case where it is appropriate to update some of the simulator random variables in an internal SMC, where others are simulated independently for each parameter (as described in Sect. 3.3.5).

Various random graph models have been proposed to model the formation of complex networks in biology. In particular in the study of protein interaction networks, growth models where networks are built through the addition of nodes over many steps, have been considered, such as linear preferential attachment models (Barabási and Albert 1999), producing scale-free networks, and biologically inspired node duplication models (Vázquez et al. 2003; Pastor-Satorras et al. 2003). It has been shown that the final structure of some of these models can depend heavily on the unobserved initial state of the network used in the growth model (Hormozdiari et al. 2007).

We consider a duplication-divergence model of network growth (Vázquez et al. 2003; Pastor-Satorras et al. 2003), where starting from an initial seed network, the network is grown in discrete steps, selecting a node at random to duplicate at each step. The duplicated node retains the edges of the original with a probability *p*, and forms a link to the original node with probability *r*. This mimics the biological process of gene duplication, where genes are copied and then diverge in function over time, with their protein products losing some of the interactions of the original gene in the process.

**Fig. 7:**
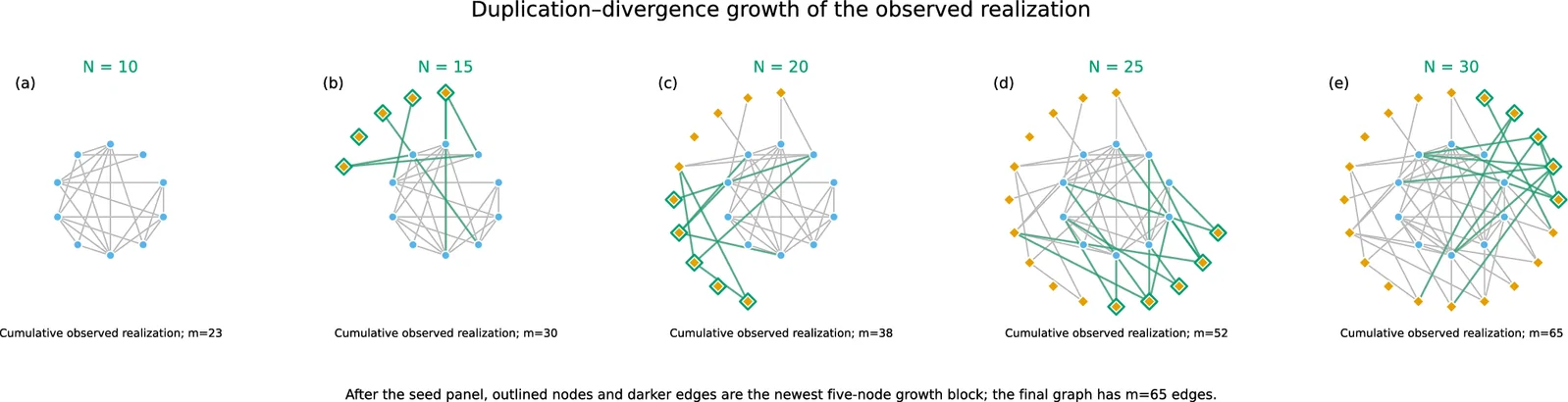
An example of the duplication-divergence process

Our aim in this section is to estimate the parameters *p* and *r* given simulated network data *y*. From a seed generated from an Erdős–Rényi graph on $d_s=20$ nodes, we then grow this with d=300 nodes using the duplication-divergence process (with parameters p=0.5 and r=0.2) as follows. At each iteration of the model, we select a node $x_i\in \mathcal {N}$ uniformly at random to duplicate. To do so we first create a new node in the network $x^*$, with no edges. Then we take all nodes $x_j\mid (x_i,x_j)\in \mathcal {E}$ neighbouring $x_i$, and attach them to the new node $x^*$ forming new edges $(x_j,x^*)$, each with probability *p*. Finally the new node $x^*$ is connected to the node that was duplicated, $x_i$, forming an edge $(x_i,x^*)$, with probability *r*. We denote the random variables used in simulating the seed network as $u_s$, and those used in duplication-divergence process as $u_r$. An illustration of this process can be found in Fig. 7 and a mathematical description is in Appendix D.

**Fig. 8:**
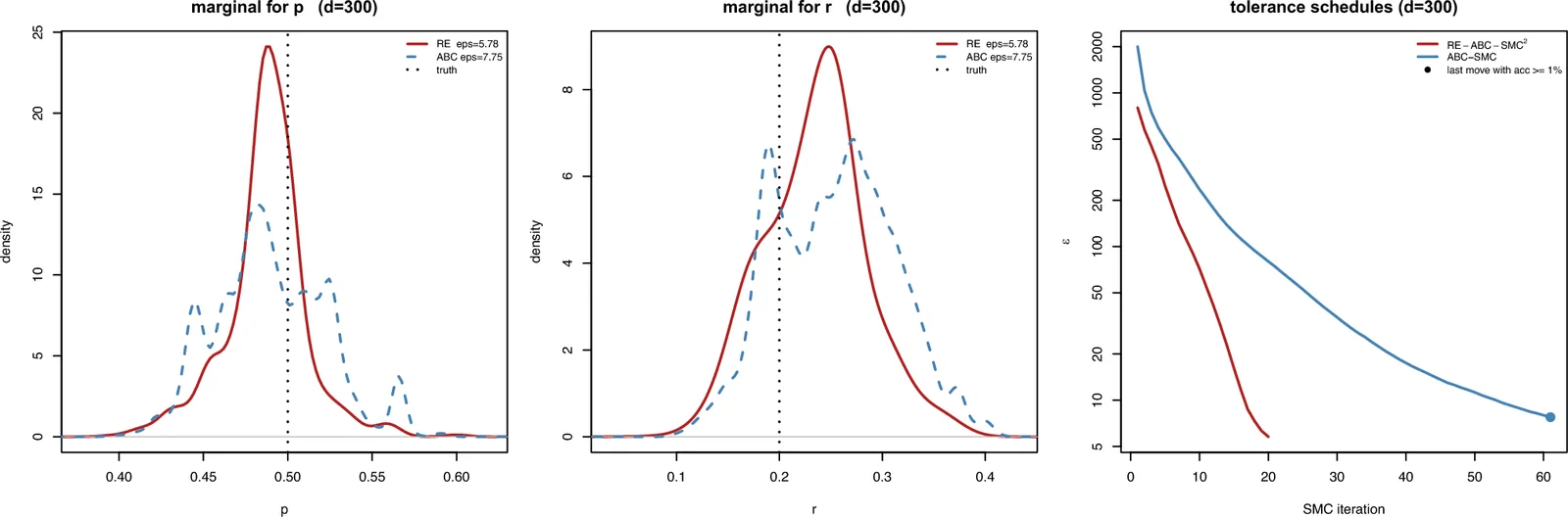
Estimated marginal posterior distributions and adaptive tolerance schedules for ABC-SMC and RE-ABC SMC$^2$ on the duplication-divergence random graph model

fv To apply the RE-ABC-SMC$^{2}$ methodology in this context, we use the idea in Sect. 3.3.5 of dividing the *u* variables into two parts: the $u_s$ variables will be updated using MCMC moves in the internal SMC, and the $u_r$ variables will not be updated since the trans-dimensional space would be difficult to update effectively using MCMC.

We used the uniform prior $\mathcal {U}\left( 0, 1\right)$ for both *p* and *r*. $P_{\epsilon }$ was chosen to be the uniform distribution $P_{\epsilon }\left( y \mid x \right) = (2\epsilon )^{-1}$ for $d(y,x) \le \epsilon$ and 0 otherwise, where *d*(*y*, *x*) is an approximation to the edit distance between the two graphs *y* and *x*. The edit distance is defined as the smallest number of edges that would need to either be added to or deleted from *y* or *x* for the two graphs to become isomorphic. This is prohibitively computationally expensive to calculate directly, but can be approximated as in Thorne and Stumpf (2012) using the ordered eigenvalues $\alpha _1,\ldots ,\alpha _d$ and $\beta _1,\ldots ,\beta _d$ of the adjacency matrices of *y* and *x* respectively as

$$ d(y,x) \approx \sum _i (\alpha _i-\beta _i)^2. $$

To construct an MCMC kernel on $u^s$, we apply a Metropolis-Hastings sampler with a proposal that either adds or deletes an edge in the seed network with equal probability $q_{add}=q_{del}=0.5$. When an edge addition proposal is chosen, one of the $N(N-1)/2-|\mathcal {E}|$ possible pairs of unconnected nodes is selected uniformly at random, and an edge added between them. For an edge deletion proposal, one of the $|\mathcal {E}|$ edges in the seed network is chosen uniformly at random and deleted. For the move step on θ, we used a Metropolis-Hastings algorithm with a truncated (from 0 to 1) Gaussian proposal with variance chosen to be equal to the variance of the weighted particles after the reweighting step. The number of iterations of the MCMC was again determined adaptively as described in Sect. 3.3.3. In all SMC algorithms again we resample when the ESS drops below the proportion α=0.5 of the number of particles, and we chose the sequence of $\epsilon _t$ adaptively such that $\epsilon _t$ is as close as possible to giving a CESS of $\beta N_{\theta }$, where β=0.9.

We used $N_{\theta } = 200$ and $N_u=100$ in our run of RE-ABC-SMC$^2$, with and $N_\theta =20000$ and $N_x=1$ for ABC-SMC. The results for the ABC-SMC run are given for the target where the acceptance rate of the MCMC moves on θ reduced to less than 0.01 (after this point the particles become increasingly degenerate). Figure 8 gives a comparison of the estimated marginal and the adaptive schedules found using ABC-SMC and RE-ABC-SMC$^2$. Again we see that for RE-ABC-SMC$^2$ the tolerance decreases more quickly. In this example RE-ABC-SMC$^2$ does not outperform ABC-SMC as dramatically as in some of our other examples, but we observe degeneracy in the ABC-SMC posterior that is not present for RE-ABC-SMC$^2$.

## Conclusions

This paper builds on the particle MCMC methodology of Prangle et al. (2018), introducing an SMC counterpart to the approach in that paper. The advantage of the Prangle et al. (2018) approach over standard ABC techniques is that it uses an SMC, rather than an IS, likelihood estimator. IS requires an exponential number of points in the dimension of the data to control the variance of the likelihood estimator, rendering ABC impracticable unless the dimension of the data is reduced by considering only summary statistics. The rare event SMC approach requires a cubic number of points in the dimension of the data, making it possible to use ABC on larger data sets without taking summary statistics. The new SMC$^2$ methodology in this paper inherits these advantageous properties, whilst also inheriting the useful properties of the widely-used ABC-SMC algorithms: for example that it requires few tuning parameters, due to the adaptive techniques outlined in Sect. 3.3; and that the model evidence may be estimated directly from the SMC output (Didelot et al. 2011).

At the core of the approach is the use of the reparameterisation trick described in Sect. 2. The real significance of this idea is that it removes the intractable likelihood from ABC, enabling one to use any available Bayesian computation technique for inferring the joint posterior of θ and *u*. This paper, and Prangle et al. (2018), have in common that they use a pseudo-marginal approach for this task. However, just as outside the ABC context, the most appropriate technique depends on the structure of the posterior: in some cases it may be more effective to use the HMC of Graham and Storkey (2017); in others it may be more effective to use an SMC directly on the joint posterior of θ and *u* (as in a parallel work to ours in Zhang (2022)). The use of a pseudo-marginal approach, and hence the algorithm in this paper, is effective when there is a strong posterior dependence between θ and *u* and it is not easy to construct an MCMC on this joint space, and when one has available an effective approach to estimating the marginal (with respect to *u*) likelihood of θ (as is the SMC method used in this paper).

The limits of this reparameterisation trick are that in practice it may be difficult to rewrite a simulator model in this way. Often ABC is used in the context when “unpacking the black box” simulator is problematic for practical reasons: e.g. a model is developed over several years by a researcher in an applied field and is coded in such a way that it is not practicable to recode it in such a way that the sources of randomness *u* can be isolated as inputs to the simulator. In this case, it would technically be possible to use the methods from this paper, but in practice it may require substantial effort.

In some situations it may be possible to reparameterise the model in multiple ways, and one parameterisation may lead to a more efficient algorithm than another. This is not dissimilar to the situation encountered in the design of MCMC algorithms, where reparameterising a model may lead to a more efficient algorithm, e.g. centred or non-centred parameterisations in MCMC (Papaspiliopoulos et al. 2007) or active subspaces (Ripoli and Everitt 2025). Outside of these cases it is unlikely to be obvious in advance which parameterisation is most suitable.

Another situation is the case mentioned in this paper, where the space of some of the variables $u_r$ is sufficiently complicated that cannot (currently) hope to design an MCMC move that can explore the space effectively. For these variables the situation remains the same as for the original formulation of ABC: that the only available proposal is the simulator.

The complexity of the algorithm is cubic in the dimension of the data. Therefore in many applications reducing the data to a vector of summary statistics is likely to be useful as it is in standard ABC, although when using the new approach the dimension of these statistics can be somewhat higher (the empirical results suggest d≈100 is feasible) compared to standard ABC (when usually d<10).

## Data Availability

No datasets were generated or analysed during the current study.

## A Target distribution of RE-ABC-SMC$^2$

To show that RE-ABC-SMC$^{2}$ has the desired target distribution we follow the argument in Chopin et al. (2013) We begin by examining the target distribution at the first iteration t=1, before moving on to the t≥2. We do not fully reproduce the argument in that paper: instead we explain the main principles; enough to illustrate the equivalence between the two situations.

At iteration *t* we require our algorithm to have as one of its marginals the target distribution

$$ \pi _{t,\theta ,u}\left( \theta ,u\mid y\right) =\frac{p\left( \theta \right) \phi \left( H\left( u,\theta \right) \right) P_{\epsilon _{t}}\left( y\mid x\right) }{p_{t}\left( y\right) }, $$

which itself has marginal distribution

$$ \pi _{t,\theta }\left( \theta \mid y\right) =\frac{p\left( \theta \right) l_{t}\left( y\mid \theta \right) }{p_{t}\left( y\right) }, $$

where

$$ l_{t}\left( y\mid \theta \right) =\int _{u}P_{\epsilon _{t}}\left( y\mid x\right) \phi \left( H\left( u,\theta \right) \right) du. $$

At t=0, $\left\{ \theta _{0}^{m}\right\} _{m=1}^{N_{\theta }}\sim p$ and $\left\{ u_{0}^{n,m}\right\} _{n=1}^{N_{u}}\sim \phi \left( \cdot \mid \theta _{0}^{m}\right)$ for $m=1:N_{u}$. Thus we write the target at iteration 0 as

$$ p\left( \theta \right) \prod _{n=1}^{N_{u}}\phi \left( u_{0}^{n}\mid \theta \right) . $$

At t=1 each particle is reweighted using the unbiased likelihood estimator

9 $$\begin{aligned} \hat{l}_{1}\left( y\mid \theta \right) =\frac{1}{N_{u}}\sum _{n=1}^{N_{u}}P_{\epsilon _{1}}\left( y\mid H\left( u_{0}^{n},\theta \right) \right) . \end{aligned}$$

We denote the distribution of the variables generated at iteration 0 used to estimate the likelihood at iteration 1 as $\psi _{0}\left( \left\{ u_{0}^{n}\right\} _{n=1}^{N_{u}}\mid \theta \right) =\prod _{n=1}^{N_{u}}\phi \left( u_{0}^{n}\mid \theta \right)$. The weight update in Eq. (9) results from the target distribution at t=1 being

10 $$\begin{aligned} \pi _{1}\left( \theta ,\left\{ u_{0}^{n}\right\} _{n=1}^{N_{u}}\right) =p\left( \theta \right) \psi _{0}\left( \left\{ u_{0}^{n}\right\} _{n=1}^{N_{u}}\mid \theta \right) \frac{\hat{l}_{1}\left( y\mid \theta \right) }{p_{1}\left( y\right) }, \end{aligned}$$

with the normalising constant following from the unbiasedess of Eq. (9). We can rewrite this target as

11 $$\begin{aligned} &  \pi _{1}\left( \theta ,\left\{ u_{0}^{n}\right\} _{n=1}^{N_{u}}\right) \nonumber \\ &  \quad = \frac{p\left( \theta \right) }{p_{1}\left( y\right) }\prod _{n=1}^{N_{u}}\phi \left( u_{0}^{n}\mid \theta \right) \nonumber \\ &  \quad \quad \times \left( \frac{1}{N_{u}}\sum _{n=1}^{N_{u}}P_{\epsilon _{1}}\left( y\mid H\left( u_{0}^{n},\theta \right) \right) \right) \nonumber \\ &  \quad \frac{1}{N_{u}}\sum _{n=1}^{N_{u}}\frac{p\left( \theta \right) }{p_{1}\left( y\right) }\phi \left( u_{0}^{n}\mid \theta \right) P_{\epsilon _{1}}\left( y\mid H\left( u_{0}^{n},\theta \right) \right) \nonumber \\ &  \quad \quad \times \left( \prod _{\begin{array}{c} i=1\\ i\ne n \end{array} }^{N_{u}}\phi \left( u_{0}^{i}\mid \theta \right) \right) \nonumber \\ &  \quad \frac{\pi _{1,\theta }\left( \theta \mid y\right) }{N_{u}}\sum _{n=1}^{N_{u}}\pi _{1,u}\left( u_{0}^{n}\mid y,\theta \right) \nonumber \\ &  \quad \quad \times \left( \prod _{\begin{array}{c} i=1\\ i\ne n \end{array}}^{N_{u}}\phi \left( u_{0}^{i}\mid \theta \right) \right) \end{aligned}$$

where we used the result

$$\begin{aligned} &  p\left( \theta \right) \phi \left( u_{0}^{n}\mid \theta \right) P_{\epsilon _{1}}\left( y\mid H\left( u_{0}^{n},\theta \right) \right) \\ &  \quad =p_{1}\left( y\right) \pi _{t,\theta }\left( \theta \mid y\right) \pi _{t,u}\left( u_{0}^{n}\mid y,\theta \right) . \end{aligned}$$

Equation (11) includes in its marginals the target at iteration 1 of $\pi _{1,\theta ,u}\left( \theta ,u\mid y\right) =\pi _{1,\theta }\left( \theta \mid y\right) \pi _{1,u}\left( u_{0}^{n}\mid y,\theta \right)$.

For t≥2, similarly to Eq. 10, we again have that our target distribution is defined to be the prior, multiplied by the likelihood estimate, multipled by the distribution of the variables used in the likelihood estimator, multiplied by the normalising constant (which again follows from the unbiasedness of the likelihood estimator)

12 $$\begin{aligned} \pi _{t}\bigl (\theta ,\{u_{0:t-1}^{n},a_{0:t-1}^{n}\}\bigr )&=p(\theta )\,\psi _{t-1}\bigl (\{u_{0:t-1}^{n},a_{0:t-1}^{n}\}\mid \theta \bigr )\nonumber \\&\quad \times \frac{\hat{l}_{t}(y\mid \theta )}{p_{t}(y)}\,. \end{aligned}$$

where $\psi _{t-1,\theta }$ is the distribution of all of the random variables generated by the internal SMC up to time *t*. Similarly to the t=1 case, we may rearrange Eq. (13) to see that $\pi _{t,\theta ,u}\left( \theta ,u\mid y\right)$ is included in its marginals:

13 $$\begin{aligned} \begin{aligned} \pi _{t}\bigl (\theta ,\{u_{0:t-1}^{n},a_{0:t-1}^{n}\}\bigr )&= \frac{\pi _{t,\theta }(\theta \mid y)}{N_{u}} \sum _{n=1}^{N_{u}} \frac{\pi _{t,u}(\textbf{u}_{1:t-1}^{n}\mid y,\theta )}{N_{u}^{\,t-1}}\\&\qquad \times \prod _{\begin{array}{c} i=1\\ i\ne \textbf{h}_{t}^{n}(0) \end{array}}^{N_{u}} \phi (u_{0}^{i}\mid \theta )\\&\qquad \times \prod _{s=2}^{t} \prod _{\begin{array}{c} i=1\\ i\ne \textbf{h}_{t}^{n}(s-1) \end{array}}^{N_{u}} w_{s-1}^{a_{s-1}^{i}}\\&\qquad \,\times K_{s-1,u}\bigl (u_{s-1}^{i}\mid u_{s-2}^{a_{s-2}^{i}},\theta \bigr )\,. \end{aligned} \end{aligned}$$

where $\textbf{u}_{1:t-1}^{n}$ and $\textbf{h}_{t}^{n}$ are deterministic functions of $\left\{ u_{0:t-1}^{n}\right\} _{n=1}^{N_{u}}$ and $\left\{ a_{0:t-1}^{n}\right\} _{n=1}^{N_{u}}$ defined as in Chopin et al. (2013): $\textbf{h}_{t}^{n}=\left( \textbf{h}_{t}^{n}\left( 0\right) ,...,\textbf{h}_{t}^{n}\left( t-1\right) \right)$ denotes the index history of $u_{t-1}^{n}$, i.e. $\textbf{h}_{t}^{n}\left( t-1\right) =n$ and $\textbf{h}_{t}^{n}\left( s\right) =a^{\textbf{h}_{t}^{n}\left( s+1\right) }$, recursively for s=t-2,...,0, and $\textbf{u}_{1:t-1}^{n}=\left( \textbf{u}_{1:t-1}^{n}(0),...,\textbf{u}_{1:t-1}^{n}(t-1)\right)$ denotes the state trajectory of particle $u_{t-1}^{n}$, i.e. $\textbf{u}_{1:t-1}^{n}(s)=u_{s}^{h_{t}^{n}(s)}$, for s=0,...,t-1.

The rearrangement of this target follows exactly the argument in Chopin et al. (2013), so is not reproduced here. The one difference in the notation from that paper is that here the index of the *u* variable is one fewer than in Chopin et al. (2013): i.e. Eq. (13) uses $\psi _{t-1}\left( \left\{ u_{0:t-1}^{n},a_{0:t-1}^{n}\right\} _{n=1}^{N_{u}}\mid \theta \right)$, whereas the equivalent term in Chopin et al. (2013) would be $\psi _{t}\left( \left\{ u_{1:t}^{n},a_{1:t-1}^{n}\right\} _{n=1}^{N_{u}}\mid \theta \right)$. The reason is that since our internal SMC involves an MCMC move, the weight update in the internal SMC only involves the values of the particles from the previous iteration. In Chopin et al. (2013), the internal SMC is a particle filter, where the weight update uses the values of the internal particles proposed at iteration *t*.

## B Preconditioned Crank–Nicolson MALA for the internal MCMC

### B.1 MCMC move

The internal rare-event SMC uses an MCMC move on its *u*-particles with an MCMC kernel that leaves the current internal target invariant. Let $L_{\epsilon }(u)=P_{\epsilon }\!\bigl (y\mid H(u,\theta )\bigr )$ be the ABC kernel viewed as a likelihood in *u*. For a fixed parameter θ the conditional posterior $\pi _{\epsilon }(u)\ \propto \ L_{\epsilon }(u)\,\phi (u)$ can be seen as a change of measure from ϕ, which when ϕ s Gaussian $\phi (u)=\mathcal {MVN}(u;0,I_{d})$ permits the use of a preconditioned Crank–Nicolson (pCN) MCMC move. A standard random-walk or Metropolis-adjusted Langevin (MALA) proposal would require a step size shrinking with *d* to keep a non-vanishing acceptance rate, whereas a pCN move avoids this by discretising the ϕ-invariant Langevin dynamics

$$\begin{aligned} \textrm{d}U_{t}= -\tfrac{1}{2} U_{t}\,\textrm{d}t + \nabla \!\log L_{\varepsilon }(U_{t})\,\textrm{d}t + \textrm{d}W_{t} \end{aligned}$$

with the Crank–Nicolson scheme, which yields a MCMC kernel whose mixing is robust to *d* (Cotter et al. 2013).

Let $g_{\varepsilon }(u):=\nabla _{u}\log L_{\varepsilon }(u)$ for the gradient of the log ABC kernel. Given a step size δ>0 set

14 $$\begin{aligned} \begin{gathered} a=\frac{1-\delta /4}{1+\delta /4},\qquad b=\frac{\sqrt{\delta }}{1+\delta /4},\qquad c=\frac{\delta /2}{1+\delta /4},\\ \text {so that}\quad a^{2}+b^{2}=1 . \end{gathered} \end{aligned}$$

The proposal from the current state *u* is

$$\begin{aligned} u^{\star }= \underbrace{a\,u + c\,g_{\varepsilon }(u)}_{=:~m(u)} \;+\; b\,\xi , \qquad \xi \sim \mathcal {N}(0,I_{d}), \end{aligned}$$

i.e. a Gaussian proposal $q(\cdot \mid u)=\mathcal {N}\!\bigl (m(u),\,b^{2}I_{d}\bigr )$ with a Langevin drift $c\,g_{\varepsilon }(u)$ superimposed on the pCN mean au. The identity $a^{2}+b^{2}=1$ is what makes the drift-free (c=0) map ϕ-reversible.

The log acceptance ratio for this proposal is given by

$$\begin{aligned} {\begin{matrix} \log r ={}& \underbrace{\log L_{\varepsilon }(u^{\star })-\log L_{\varepsilon }(u)}_{\text {kernel ratio}}\\ & +\underbrace{\log \phi (u^{\star })-\log \phi (u)}_{-\frac{1}{2}(\Vert u^{\star }\Vert ^{2}-\Vert u\Vert ^{2})}\\ & +\underbrace{\frac{\Vert u^{\star }-m(u)\Vert ^{2}-\Vert u-m^{\star }\Vert ^{2}}{2b^{2}}}_{\log q(u\mid u^{\star })-\log q(u^{\star }\mid u)}. \end{matrix}} \end{aligned}$$

### B.2 Adaptive scheme

The step size δ (stored per inner SMC and persisting across tolerance steps) is adapted after each sweep towards the optimal Langevin acceptance rate 0.574,

$$\begin{aligned} \delta \leftarrow \operatorname {min}\!\Bigl (\delta \,\exp \bigl \{\tfrac{1}{2}(\hat{p}-0.574)\bigr \},\ 10^{-6},\ 3.99\Bigr ), \end{aligned}$$

where $\hat{p}$ is the empirical acceptance of the sweep; setting δ<4 so a∈(-1,1]. The number of sweeps per SMC step is itself chosen adaptively so that a target fraction of particles is moved as in Sect. 3.3.3.

## C Gradient of the ABC kernel for the Lotka–Volterra example

The pCN–MALA move on the noise *u* in the Euler-Maruyama solver for the Lotka-Volterra model requires the gradient of the log ABC kernel with respect to *u*. This gradient is computed by reverse-mode differentiation of the solver, at the cost of a single forward solve followed by a single backward sweep. This appendix derives that recursion and states the resulting algorithm.

In section 4.3 we chose the ABC kernel is Gaussian, so its logarithm is

15 $$\begin{aligned} \begin{aligned} \ell (u)&:= \log K_\varepsilon \bigl (y\mid H(u,\theta )\bigr )\\&\propto -\frac{1}{2\varepsilon ^2}\,\bigl \Vert H(u,\theta )-y\bigr \Vert ^2\\&= -\frac{1}{2\varepsilon ^2}\sum _{i=1}^{d}\bigl \Vert x_{i \times m}-y_i\bigr \Vert ^2 , \end{aligned} \end{aligned}$$

where $y_t$ is the observation at time *t* (the first term is omitted since $x_0=y_0$). We require $\nabla _u\ell (u)$ to construct the pCN–MALA proposal for use in the internal MCMC move with target $K_\varepsilon (y\mid H(u,\theta ))\,\phi (u)$.

Differentiating (7) with respect to the incoming state and the noise gives the two blocks needed by the chain rule. For the noise,

16 $$\begin{aligned} B_k:=\frac{\partial \tilde{x}_k}{\partial u_k} = \sqrt{\Delta t}\;S\,\operatorname {diag}\!\bigl (\sqrt{h_{k-1}}\bigr )\in \mathbb {R}^{2\times 3}. \end{aligned}$$

For the state, note that the *i*-th reaction contributes $h_{k-1}^{(i)}\Delta t+\sqrt{h_{k-1}^{(i)}}\sqrt{\Delta t}\,u_k^{(i)}$, whose derivative with respect to $h^{(i)}$ is the scalar

17 $$\begin{aligned} g_k^{(i)}:=\Delta t+\frac{\sqrt{\Delta t}\,u_k^{(i)}}{2\sqrt{h_{k-1}^{(i)}}}, \end{aligned}$$

so that

18 $$\begin{aligned} A_k:=\frac{\partial \tilde{x}_k}{\partial x_{k-1}} = I_2 + S\,\operatorname {diag}(g_k)\,J_h(x_{k-1};\theta ). \end{aligned}$$

Combining with the boundary map (8) yields the full one-step Jacobians $\partial x_k/\partial x_{k-1}=D_kA_k$ and $\partial x_k/\partial z_k=D_kB_k$, where

19 $$\begin{aligned} D_k:=\operatorname {diag}(d_k), \qquad d_k^{(i)}={\left\{ \begin{array}{ll} 0, & \text {when to { c},}\\ -1, & \text {reflection } \tilde{x}_k^{(i)}<0 \text {,}\\ +1, & \text {otherwise.} \end{array}\right. } \end{aligned}$$

Define $\lambda _k:=\nabla _{x_k}\ell$ which accumulates both the observation term at step *k* and all contributions propagated back from steps k+1,⋯,K. Because $u_k$ influences ℓ only through $x_k$ and its descendants,

20 $$\begin{aligned} \nabla _{z_k}\ell =\Bigl (\tfrac{\partial x_k}{\partial z_k}\Bigr )^{\!\top }\lambda _k =B_k^\top D_k\lambda _k . \end{aligned}$$

Using the definition $\mu _k:=D_k\lambda _k$, from (16) and $D_k=D_k^\top$, we have that our recursion is given by

21 $$\begin{aligned} \nabla _{z_k}\ell =B_k^\top \mu _k =\sqrt{\Delta t}\,\operatorname {diag}\!\bigl (\sqrt{h_{k-1}}\bigr )\,S^\top \mu _k\; \end{aligned}$$

and

22 $$\begin{aligned} \lambda _{k-1}=A_k^\top \mu _k =\mu _k+J_h(x_{k-1};\theta )^\top \operatorname {diag}(g_k)\,S^\top \mu _k . \end{aligned}$$

The observation terms in (15) enter the recursion at the time steps corresponding to the measurements: whenever k=t×m for some $t\in \{1,\dots ,T\}$,

23 $$\begin{aligned} \lambda _k \mathrel {+}= -\frac{1}{\varepsilon ^2}\bigl (x_{tm}-y_t\bigr ), \end{aligned}$$

before forming $\mu _k$. The recursion is initialised with $\lambda _K$ set to the term (23) at k=K (and 0 otherwise), with the and the gradient being is $\nabla _u\ell =(\nabla _{u_1}\ell ^\top ,\dots ,\nabla _{u_K}\ell ^\top )^\top$ after implementing the recursion. This is summarised in Algorithm 6.

## D Description of the duplication-divergence model

Let θ=(p,r) and *x* denote the simulated graph on *d* nodes. First a seed graph is simulated followed by the duplication-divergence phase. The randomness of each is isolated in separate blocks $u_s$ and $u_r$ respectively. $u_s$ and $u_r$ consist of independent $\mathcal {U}(0,1)$ variates, so $\phi (u)=\mathcal {U}\!\left( [0,1]^{\dim u}\right)$.

The seed is an Erdős–Rényi graph on $d_s=20$ nodes $\mathcal {N}_0=\{1,\dots ,d_s\}$ in which each of the $$M_s=\left( {\begin{array}{c}d_s\\ 2\end{array}}\right) $$ possible edges is present independently with probability *a*. Precisely, enumerate the possible seed edges $e=1,\dots ,M_s$ and set

$$\begin{aligned} u_s=(u_{s,1},\dots ,u_{s,M_s})\in [0,1]^{M_s},\qquad u_{s,e}\overset{\text {iid}}{\sim }\mathcal {U}(0,1). \end{aligned}$$

The seed graph is the deterministic function $G_0=S(u_s)=(\mathcal {N}_0,\mathcal {E}_0)$,

$$\begin{aligned} \mathcal {E}_0 = \bigl \{\, e \;:\; u_{s,e} < a \,\bigr \}. \end{aligned}$$

The graph is grown from $d_s$ to *d* nodes by $K=d-d_s$ duplication steps. Define

$$\begin{aligned} u_r=(u_{r,1},u_{r,2},\dots )\overset{\text {iid}}{\sim }\mathcal {U}(0,1), \end{aligned}$$

whose total length is itself random, for the reason made explicit below. Given the current graph $G_{k-1}=(\mathcal {N}_{k-1},\mathcal {E}_{k-1})$ with $|\mathcal {N}_{k-1}|=d_s+k-1$ nodes, step *k* (k=1,⋯,K) reads the next few uniforms and forms $G_k$ as follows.

1. *Duplication*. Use one uniform *u* and select the node to duplicate uniformly at random, $i_k=\bigl \lceil (d_s+k-1)\,u\bigr \rceil$; create a fresh node $\nu _k=d_s+k$ with no edges.
2. *Divergence*. Let $x_{j_1},\dots ,x_{j_{m_k}}$ be the $m_k=\deg _{G_{k-1}}(i_k)$ neighbours of $i_k$. For each $\ell =1,\dots ,m_k$ use one uniform $u^{(\ell )}$ and add the edge $(x_{j_\ell },\nu _k)$ iff $u^{(\ell )}<p$.
3. *Link to parent*. Use one uniform *u* and add the edge $(i_k,\nu _k)$ iff u<r.

Denote this deterministic step map by $\Phi ^{(k)}_\theta$, so $G_k=\Phi ^{(k)}_\theta (G_{k-1})$ (a function of $G_{k-1}$ and the length-$\tau _k$ slice of $u_r$ it consumes). Step *k* uses

$$\begin{aligned} \tau _k \;=\; \underbrace{1}_{\text {select }i_k} +\underbrace{m_k}_{\text {divergence}} +\underbrace{1}_{\text {parent link}} \;=\; m_k+2 \end{aligned}$$

uniforms, and $m_k=\deg _{G_{k-1}}(i_k)$ is random: it depends on which node is selected and on the entire history of earlier growth (and seed) decisions. Hence the total length of $u_r$,

$$\begin{aligned} L \;=\; \sum _{k=1}^{K}\tau _k \;=\; \sum _{k=1}^{K}\bigl (m_k+2\bigr ), \end{aligned}$$

is a random variable, and the meaning of a given component of $u_r$ (which edge, or which node-selection, it controls) is itself determined by the preceding components. This variable, history-dependent structure is why $u_r$ is *not* updated by MCMC and is refreshed only by re-simulation.

Collecting the two phases, the simulator is the deterministic map

24 $$\begin{aligned} &  x = H(u,\theta ) = \Bigl (\Phi ^{(K)}_\theta \circ \Phi ^{(K-1)}_\theta \circ \cdots \circ \Phi ^{(1)}_\theta \circ S\Bigr )(u),\nonumber \\ &  u=(u_s,u_r). \end{aligned}$$

The variable $u=(u_s,u_r)$ is split as in the general construction: $u_s$ has the fixed dimension $M_s$ and drives the seed, and is updated by the internal MCMC; $u_r$ has the random, history-dependent dimension *L* and is left unmoved, being regenerated only when a fresh graph is simulated.
